# Rules Describing CO_2_ Activation on Single-Atom Alloys from DFT-Meta-GGA Calculations and Artificial Intelligence

**DOI:** 10.1021/acscatal.4c07178

**Published:** 2025-02-04

**Authors:** Herzain I. Rivera-Arrieta, Lucas Foppa

**Affiliations:** The NOMAD Laboratory at the Fritz Haber Institute of the Max Planck Society, Faradayweg 4-6, Berlin D-14195, Germany

**Keywords:** single-atom alloys, CO_2_ activation, artificial intelligence, subgroup discovery, materials screening, DFT simulations

## Abstract

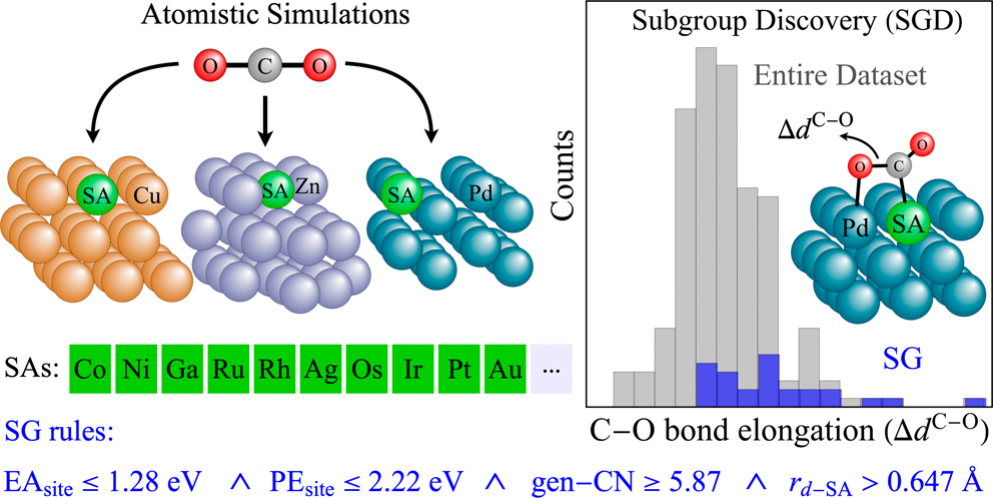

Single-atom alloys (SAAs) arise as a promising concept for the design of improved CO_2_ hydrogenation catalysts. However, from the immense number of possible SAA compositions and structures, only a few might display the properties required to be useful catalysts. Thus, the direct, high-throughput screening of materials is inefficient. Here, we use artificial intelligence to derive rules describing surface sites of SAAs that provide an effective CO_2_ activation, a crucial initial step to convert the molecule into valuable products. We start by modeling the CO_2_ interaction with 780 sites of flat and stepped surfaces of SAAs composed by Cu, Zn, and Pd hosts via high-quality DFT-mBEEF calculations. Then, we apply subgroup discovery to determine constraints on key physicochemical properties, out of 24 offered candidate descriptive parameters, characterizing subgroups (SGs) of surface sites where chemisorbed CO_2_ displays large elongations of its C–O bonds. The key identified parameters are free-atom properties of the elements constituting the surface sites, such as their electron affinity, electronegativity, and radii of the d-orbitals. Additionally, the generalized coordination number is selected as a key geometrical parameter. The SG rules are applied to identify promising surface sites from a candidate space of over 1500 possible ones in different single-atom and dual-atom alloys. Some of the promising alloys predicted by the SG rules were explicitly tested by additional DFT-mBEEF calculations and confirmed to provide a significant CO_2_ activation.

## 1 Introduction

Carbon dioxide is the main byproduct of fossil fuels combustion and the largest contributor to global warming.^1−3^ Among greenhouse gases, CO_2_ is responsible for approximately 66% of Earth’s total radiative forcing.^4^ Thus, there is an urgent need for developing chemical processes to capture and transform CO_2_ into valuable compounds, such as olefins and alcohols.^5−12^ These processes can support a sustainable society if they are combined with the large-scale production of hydrogen from renewable energy sources.^13−15^ Because the molecule is rather inert, the efficient conversion of CO_2_ requires the use of catalysis. For instance, transition-metal-based catalysts can enable the hydrogenation of CO_2_ to methanol via thermal processes.^16−20^ Currently, Cu/ZnO/Al_2_O_3_ is the state-of-the-art catalyst for performing this reaction.^21^ However, this catalyst experiences deactivation in the presence of high concentrations of water within the reaction mixture.^20−22^ Hence, developing new, water-tolerant catalysts is crucial to achieve industrial CO_2_ utilization. Pd-based CO_2_ hydrogenation catalysts are more water-tolerant than those based on Cu. Nevertheless, these systems favor the reverse water–gas shift reaction producing significant amounts of carbon monoxide.^17,18^ It has been shown that the selectivity to the desired methanol product can be increased by alloying Pd with a second metal, such as Zn or Ga.^20,23^ Therefore, alloy materials offer a promising strategy for improving the performance of CO_2_ hydrogenation catalysts.

Single-atom catalysts are a frontline approach in catalysis research.^24,25^ In these systems, isolated atoms of a metal are dispersed and stabilized on a host material. Single-atom alloys (SAAs) are metallic systems in which one single atom of a first chemical element is embedded in a monometallic host surface of a second element.^26−30^ We denote these systems hereafter as SA@host. SAAs can display unique electronic properties compared to monometallic systems or stoichiometric alloys.^31−34^ These properties can be exploited to design new catalysts. Particularly, the adsorption of reactive species on the materials surface can be modulated by the choice of the SA and host elements, and by the geometry of the adsorption sites containing the SA. Several methods for the synthesis of SAAs and applications in catalysis have been reported.^35,36^ SAAs are typically designed by embedding a highly active SA on the surface of a less active host element. This is done to enable the desired reaction while avoiding undesirable side (competing) reactions that would occur in systems of pure, highly active metals. Thus, the selectivity can be improved. Additionally, it has been shown that SAAs can display enhanced stability during time on stream (operation).^27,37^ However, due to the practically infinite compositional and structural space, designing SAAs for catalysis is a formidable challenge. Theoretical approaches like those based on density functional theory (DFT),^38^ can elucidate specific processes related to reactivity, such as surface reaction elementary steps.^39^ For instance, it is possible to model the CO_2_ activation, the key initial step for converting the molecule into valuable products.^40,41^ DFT studies also modelled the full reaction path for the CO_2_ conversion, on SAAs, to different products like methanol and ethylene.^42,43^

Artificial Intelligence (AI) can accelerate the discovery of promising materials by identifying correlations and patterns in data.^44^ Nonetheless, only few SAAs might present the properties required for them to be useful in a specific application. This is often the case in catalysis, where only a handful of compounds are known as efficient catalysts (active, selective, and stable during operation). Global AI models, such as artificial neural networks, kernel ridge regression, or Gaussian processes, might overlook these exceptional cases, since these models are designed to describe as many materials as possible, *i.e.*, to have the best predictive performance *on average* for most materials, but not necessarily the useful ones.^45,46^ Furthermore, many of these approaches produce black-box models offering little to no interpretability.^47^ Alternatively, focused AI approaches can provide descriptions of specific regions of interest in the materials space. For instance, subgroup discovery (SGD) identifies local partitions of the data associated with outstanding distributions of a given target of interest.^48−50^ In particular, SGD provides rules as constraints (*e.g.*, inequalities) on the values of the key properties identifying the materials in the subset(s) of interest. These rules provide an interpretable description of the parameters influencing the property of interest. Although approaches such as decision trees also provide interpretable models, their focus on average performance might overlook interesting, high-performance situations.^50^ We note that SGD presents significant differences compared with widely used clustering algorithms. Clustering is an unsupervised method that groups data points based on similarity, without considering a target variable nor providing explicit explanations for the grouping. In contrast, rather than assigning each point to a cluster, SGD is a supervised approach that identifies outstanding subsets or SGs of data with respect to the target. SGD also identifies rules explaining why data points belong to the identified SG.

**Figure 1:**
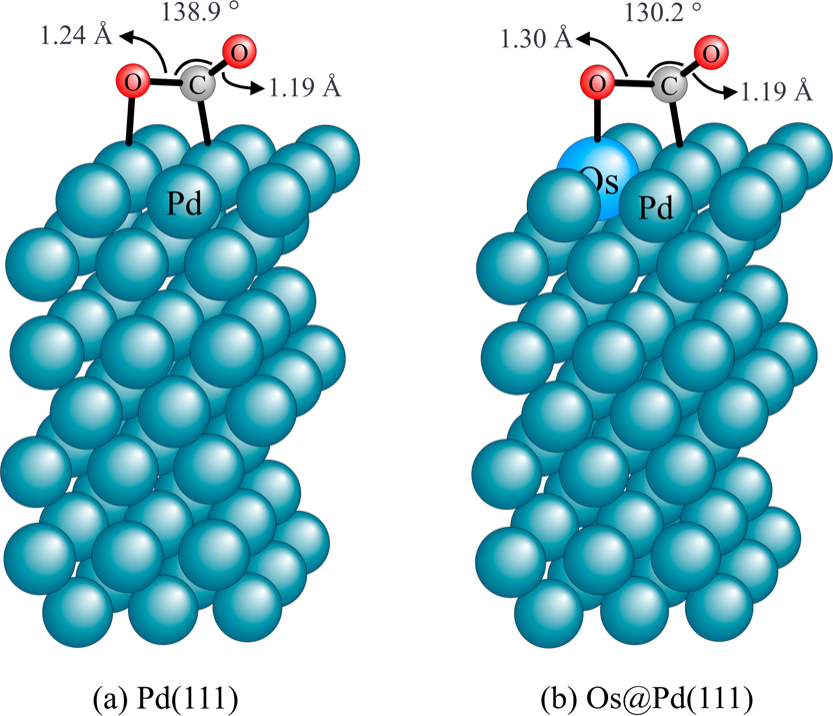
The single-atom-alloy concept, *i.e.* embedding a single atom in a host metal, can significantly affect the CO_2_ activation. (a) For the Pd(111) surface, the molecule chemisorption is not favorable ( = 0.046 eV), and the C–O bond of chemisorbed CO_2_ is slightly elongated with respect to the bond lengths in the isolated (gas-phase) molecule (1.155 Å). (b) In the Os@Pd(111) surface, CO_2_ chemisorption is favored ( = −0.337 eV) and the C–O bond is significantly elongated, indicating a stronger activation.

In this work, we combine DFT simulations with the SGD approach to obtain rules describing surface sites on SAAs that are able to activate CO_2_ effectively (Figure 1). Relying on the meta-Bayesian-error-estimation functional (mBEEF) for exchange and correlation, a semilocal meta-generalized gradient approximation (*meta*-GGA),^51^ we model the CO_2_ interaction with 780 surface sites in several flat and stepped surfaces of 36 SAAs based on Cu, Zn, and Pd host elements. We create a data set containing 24 physicochemical candidate descriptive parameters characterizing the surface sites where the molecule chemisorbs. As the target property for our AI analysis, we use the C–O bond elongation of these chemisorbed CO_2_ structures. Then, by applying SGD, we uncover descriptions of surface-site SGs in the SAAs data set resulting in a large elongation of at least one of the C–O bonds. The obtained rules highlight the key electronic and geometric properties of the SAA surface sites associated with CO_2_ activation. Based on the obtained SG rules, we efficiently identify promising alloys in a candidate space of more than 1500 possible SAAs and dual-atom alloys (DAAs).^52,53^ Through additional DFT-mBEEF calculations, we confirm the capability of the surface sites in these promising alloys to activate CO_2_. Therefore, our approach provides chemical insights into the CO_2_ activation on SAAs while enabling the efficient design of new materials.

## 2 Methodology

### 2.1 Atomistic Models of the SAA Surfaces and Calculations Settings

**Figure 2:**
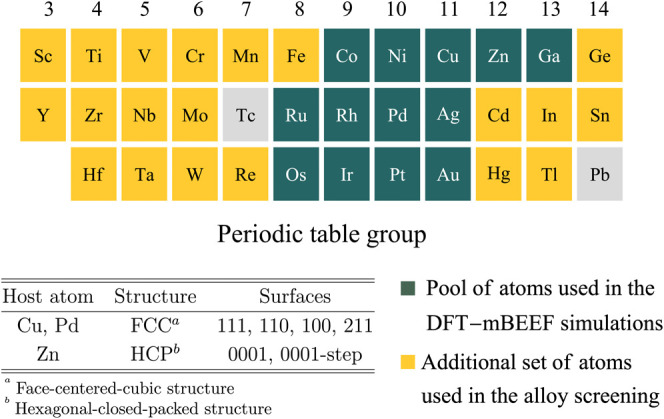
Single-atoms, hosts, and considered surface terminations in our study of the CO_2_ activation on SAAs. The structures containing Co and Ni atoms involved spin-polarized calculations.

Based on previous work on the CO_2_ hydrogenation by thermal^16−20^ and electrochemical catalysis,^54−56^ we choose Cu, Zn, and Pd as host metals in our SAAs. Twelve different SAs are combined with each of the three hosts resulting in 36 SAAs. Overall, 120 flat and stepped SAA surfaces and 780 different surface sites were considered (Figure 2). The sites in the pristine monometallic surfaces of the three host metals were also included in the study.

The DFT simulations were performed with the FHI-aims package^57^ and managed through the Atomic Simulation Environment.^58^ We use the mBEEF *meta*-GGA functional^51^ to perform our calculations. Prior benchmark work^59−61^ confirms mBEEF as an appropriate choice to model the interaction between CO_2_ and the SAA surfaces. mBEEF provides the lowest errors for bulk and surface properties in Cu, Zn, and Pd among 22 exchange–correlation functionals considered in a previous work.^59^ Moreover, mBEEF shows a better agreement with the random-phase approximation in atomic and molecular adsorption energies on different metal surfaces compared to the widely used GGA functionals PBE and RPBE.^60^ The use of the Hartree potential correction of FHI-aims allowed us to speed up the simulations. A detailed description of the slab models, considered surface sites, and the settings of the DFT-mBEEF simulations, is provided in the Supporting Information Section S1.

We evaluate the CO_2_ adsorption energy  to judge whether the interaction with the SAA surface is energetically favorable.
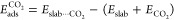
1Here,  is the energy of the slab with adsorbed CO_2_, *E*_slab_ is the energy of the clean slab (without the molecule), and *E*_CO_2__ is the energy of the isolated CO_2_ molecule.

We used two criteria for selecting structures for the SGD analysis. First, we limited the selection only to stable chemisorbed CO_2_ structures . The latter is imposed to rule out unlikely configurations compared with the molecule’s physisorption, which exhibits  between −0.16 and −0.12 eV in the considered hosts and SAA surfaces. The second selection criterion establishes that CO_2_ must display a direct interaction with the SA on the surface (see Figure 1b). As the CO_2_ chemisorption on the sites of monometallic Cu, Zn, and Pd surfaces is included in our analysis, the second criterion excludes surface adsorption sites on SAA structures that are only composed by atoms of the host element. Moreover, we do not observe significant geometry or energy differences between the CO_2_ chemisorption on sites composed only by host elements in the SAA surfaces and equivalent sites on monometallic surfaces (see Table S2 in the Supporting Information and related discussion).

Different parameters have been suggested as indicators of CO_2_ activation, being the O–C–O angle the most common one.^40^ However, atomistic simulations on model surfaces have shown that oxide materials experimentally identified as catalysts for CO_2_ conversion exhibit significant C–O bond elongation in chemisorbed CO_2_.^62^ Thus, we consider the C–O bond elongation in the chemisorbed CO_2_ molecule (Δ*d*_max_^C–O^) as the indicator of the molecule activation.^62^ This quantity is defined as
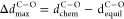
2were *d*_chem_^C–O^ is the largest distance between the two C–O bond distances in the chemisorbed CO_2_ molecule, and d_equil_^C–O^ refers to the distance between C and O in an optimized gas-phase CO_2_ molecule evaluated with DFT-mBEEF (1.155 Å).

### 2.2 Subgroup Discovery

Starting with a data set *P̃*, containing *N* physicochemical candidate descriptive parameters (φ_*i*_) and a target quantity of interest *Y*, *e.g.* a material’s property, SGD searches along the φ_*i*_-space and identifies subsets of data or subgroups (SGs) with outstanding distributions of *Y*.^48,49^ These SGs are identified by selectors (σ_*i*_) or “rules” which can be used to obtain physical insights and to screen for new materials outside the training data set. The rules typically have the form
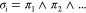
3were the π_*i*_ are propositions constraining the values of each φ_*i*_ to some minimum (π_1_ ≡ φ_1_ > *a*) or maximum (π_2_ ≡ φ_2_ < *b*) values to be determined. Usually, the number of π_*i*_ entering the description of the identified SGs is lower than 4 or 5. As not all the data points will follow each of these π_*i*_, each selector points to specific subsets in the data. When a sample in the data set follows all the propositions in a given set of rules, the sample belongs to the SG. Hence, the properties entering a selector in a SG displaying exceptional values of *Y*, are identified as the key parameter related with *Y*. The identification of the SGs is based on the maximization of a quality function *Q*
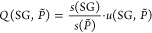
4The first term, known as the coverage, is the ratio between the size of the subgroup *s*(SG) and the size of the data set . This term prevents the selection of too small SGs. The second term is the utility function  and provides a metric for the usefulness of the SGs. In particular, our SGD study uses a two-step Monte Carlo search algorithm to identify SGs.^63^ This algorithm begins with the generation of random seed selectors (σ_seed_). Each σ_seed_ is generated with a probability proportional to . Then, these selectors are refined through an opportunistic pruning algorithm. This pruning step, involving the removal of propositions from the randomly generated σ_seed_, enables the maximization of . The target in our analysis is Δ*d*_max_^C–O^ as defined in eq 2. As we aim at identifying rules describing structures with large Δ*d*_max_^C–O^ values, we used the normalized positive mean shift function (see eq S8 in the Supporting Information). This utility function favors SGs with high mean values of Δ*d*_max_^C–O^. Hence, we should identify SGs of SAA surface sites that can significantly activate CO_2_, and weaken at least one of its bonds.

As candidate descriptive parameters, we collected 24 physicochemical properties characterizing the SAAs and the surface sites where CO_2_ chemisorbs (Table 1). These parameters are constructed considering (free-atom) properties of the elements in the alloys, of the monometallic bulk systems and of the SAA surface sites. The elemental properties are Pauling electronegativity (PE), ionization potential (IP), electron affinity (EA), and s-, p-, d-, and valence-orbital radii. The use of these parameters is based on two main considerations. First, they are accessible without the need of performing expensive computations or experiments. Second, these parameters relate to the chemical properties of elements. Thus, they might be correlated to underlying phenomena, such as the charge transfer from the surface to the molecule.^64^ We define four types of candidate descriptive parameters: host, SA, surface site, and surface site + first nearest neighbors. The host and SA parameters are properties of the elements associated with hosts and SAs. Additionally, the bulk interatomic distance is included as a host candidate parameter. The surface site and surface site + first nearest neighbors parameters are defined as the average of the elemental properties corresponding to all atoms in the site (or ensemble). In other words, if the adsorption site is composed by more than one atom, the average of the properties corresponding to all atoms in the adsorption site is taken as candidate descriptive parameter. The geometry is another factor strongly influencing the adsorbate interaction with the surface. Thus, two geometrical parameters characterizing the surface sites are included: coordination number (CN) and the generalized-CN (gen-CN).^65^ By including basic candidate descriptive parameters, we aim to derive rules capable of screening new materials efficiently, without the need of further sophisticated DFT simulations. Additional details on the SGD AI approach and on the evaluation of the candidate descriptive parameters are provided in the Supporting Information Section S3.

**Table 1:** Candidate Descriptive Parameters (φ_*i*_) Used in the Subgroup-Discovery Analysis

type	symbol	unit	description
host	PE_h_	-	host Pauling electronegativity
	IP_h_	eV	host ionization potential
	EA_h_	eV	host electron affinity
	*r*_s-h_	Å	host s-orbital atomic radius
	*r*_p-h_	Å	host p-orbital atomic radius
	*r*_d-h_	Å	host d-orbital atomic radius
	*r*_val-h_	Å	host valence radius
	bulk_h-nnd_	Å	neighbor distance in host bulk
single-atom	PE_SA_	-	SA Pauling electronegativity
	IP_SA_	eV	SA ionization potential
	EA_SA_	eV	SA electron affinity
	*r*_s-SA_	Å	SA s-orbital atomic radius
	*r*_p-SA_	Å	SA p-orbital atomic radius
	*r*_d-SA_	Å	SA d-orbital atomic radius
	*r*_val-SA_	Å	SA valence radius
surface site	PE_site_	-	surface site PE^a^
	IP_site_	eV	surface site IP^a^
	EA_site_	eV	surface site EA^a^
	site_no_	# atoms	atoms in the surface site
surface site + first neighbors	PE_snn_	-	surface site and first neighbors PE^a^
	IP_snn_	eV	surface site and first neighbors IP^a^
	EA_snn_	eV	surface site and first neighbors EA^a^
	CN	# atoms	surface site coordination number
	gen-CN	# atoms	generalized CN

a Average of all atoms in the adsorption site or ensemble.

## 3 Results and Discussion

### 3.1 DFT-mBEEF Simulations and CO_2_ Interaction with SAAs

Overall, we performed approximately 2200 simulations modeling the interaction of the CO_2_ molecule with SAAs based on the hosts Cu, Zn, and Pd. The monometallic surfaces of the hosts were also included in our analysis. Most of our simulations identified structures where CO_2_ is physisorbed. The physisorption is characterized by the molecule’s linear configuration. Besides, in the physisorbed systems, the molecule–surface distance is typically around 3 Å. Nevertheless, some simulations resulted in chemisorbed CO_2_ structures. We found 199 structures where chemisorbed CO_2_ satisfies the criteria in Section 2.1. Three kinds of interactions are identified among the structures with chemisorbed CO_2_: (a) only the carbon atom of CO_2_ bonds to the surface (η^1^), (b) the carbon and one oxygen atom of CO_2_ bond to the surface (η^2^) as in Figure 1, and (c) the three atoms of the molecule bond to the surface (η^3^). The η^1^ and η^3^ geometries are illustrated in Supporting Information Section S2.1. We analyze the correlation between Δ*d*_max_^C–O^ and  considering the overall trends observed on these 199 systems.

**Figure 3:**
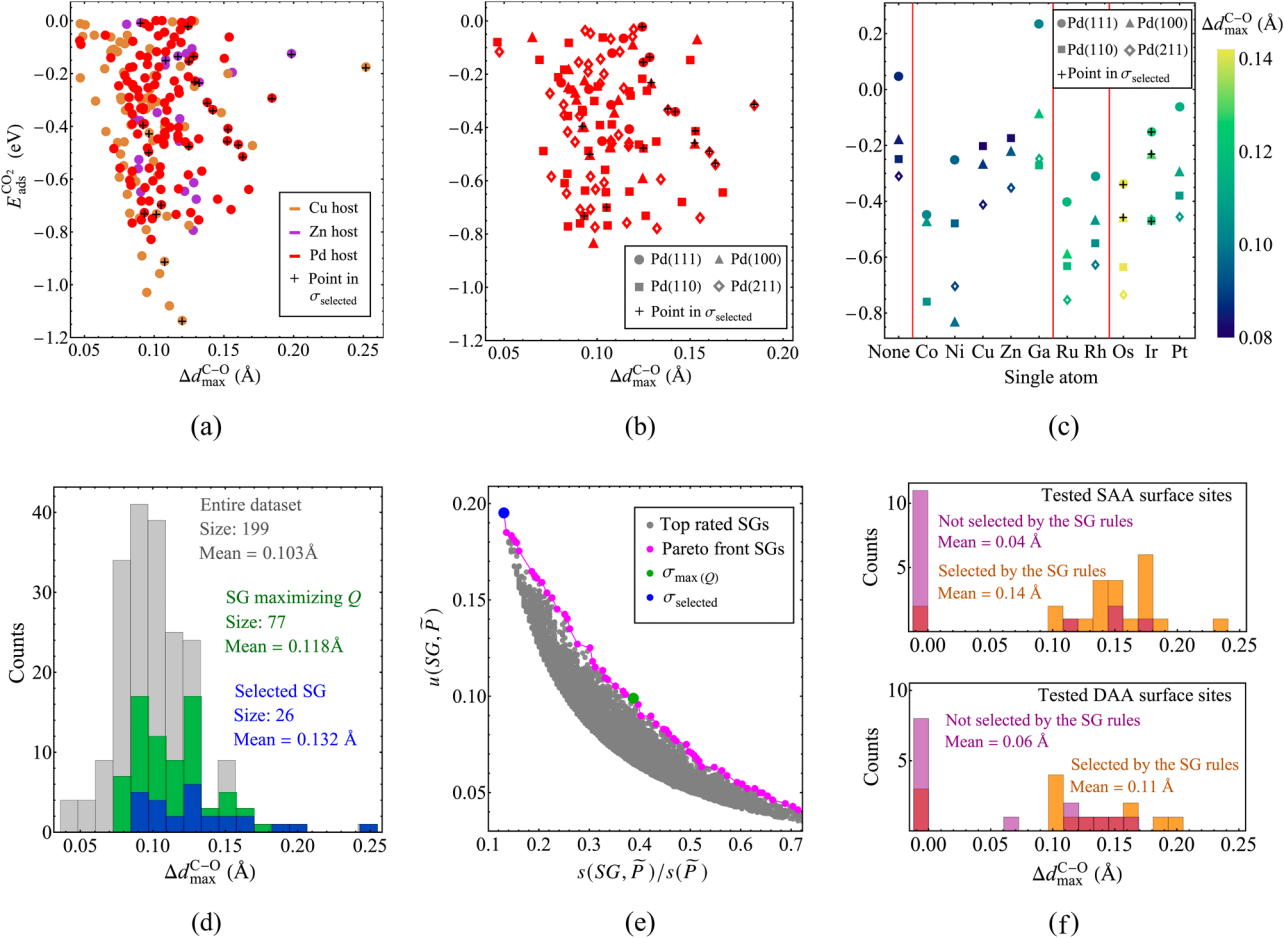
Analysis of CO_2_ chemisorption and activation on surface sites of single-atom alloys via DFT-mBEEF calculations and subgroup discovery (a) Relationship between adsorption energy  and largest C–O distance in chemisorbed CO_2_ (Δ*d*_max_^C–O^). (b) Surface-sensitivity of  and Δ*d*_max_^C–O^ for SAAs based on the Pd host. (c) SA influence on  and Δ*d*_max_^C–O^ for SAAs based on the Pd host. (d) Distribution of Δ*d*_max_^C–O^ in the entire data set and in the identified SGs σ_max(*Q*)_ and σ_selected_. (e) Top-rated SGD solutions with respect to the quality function of eq 4 and Pareto front of optimal solutions with respect to coverage (*x* axis) and normalized-positive-mean-shift utility function (*y* axis, see Supporting Information eq S8). (f) Results of new DFT-mBEEF simulations used to validate the SG rules (see details of the considered systems in Table 3).

Figure 3a shows the adsorption energy and the C–O bond elongation values associated with the structures containing chemisorbed CO_2_. The  and Δ*d*_max_^C–O^ values are in the ranges [−1.2, 0 eV] and [0.04, 0.25 Å], respectively. These wide ranges show that the surface–adsorbate adsorption energy can be largely tuned by the choice of host and SA elements and by the structure of the adsorption site. The SAAs displaying the strongest binding with the adsorbate, *i.e.*, the lowest  values, are based on the Cu host. No clear pattern can be identified among the SAAs associated with large Δ*d*_max_^C–O^ values. Figure 3a also shows that low  translates to large C–O bond elongations in some systems, implying that these quantities might be inversely correlated. However, the systems presenting the largest C–O bond elongations (Δ*d*_max_^C–O^ > 0.18 Å) display rather high CO_2_ adsorption energy ( 0.30 eV). Thus, large C–O bond elongations are not necessarily correlated with low CO_2_ adsorption energies. The analysis of the charge transfer from the SAAs surfaces to chemisorbed CO_2_ shows no clear correlation with the adsorption energy (see Supporting Information Section S2.3).

We have also verified the effect of the surface structure on the CO_2_ chemisorption. Here, we focus the discussion on surface terminations of the SAAs based on the Pd host (Figure 3b). The Pd(110) and Pd(211) terminations tend to provide SAAs with stronger CO_2_ chemisorption compared to Pd(111) and Pd(100). The Pd(110) and Pd(211) surfaces are also associated with some of the largest Δ*d*_max_^C–O^ values. These observations can be related to the fact that surface adsorption sites in (110) and (211) terminations display atoms with lower coordination (lower CN or gen-CN) compared to the (111) and (100) terminations. Thus, these more unsaturated surface atoms favor CO_2_ activation. We also notice that  is distributed across a wide range of values among the Pd(110) and Pd(211) surfaces. Thus,  does not provide a clear correlation with the molecule activation or with the surface structure for all situations considered in this work.

Figure 3c displays the influence of the SA on the  and Δ*d*_max_^C–O^. For this analysis, we focus on bridge sites of SAAs with the Pd host and corresponding to the surfaces (111), (100), (110), and (211). First, we note that the presence of the SA typically favors the CO_2_ chemisorption compared to the case of monometallic surfaces.^66^ Moreover, we observe a decrease in the  as the group of the SA increases within a given period in the periodic table. For instance, the average  decrease between Ru and Rh bridge sites in Figure 3c is 0.104 eV. In general, Δ*d*_max_^C–O^ seems to be highly influenced by the SA element. Variations in the C–O elongation among the different surface terminations are small and do not show a clear pattern. The analysis for bridge sites of SAAs based on Cu and Zn hosts (see Supporting Information Section S2.4) highlights similar trends to those for SAAs based on the Pd host.

Through the analysis of the data in Figure 3a–c, it becomes clear that the host metal, the SA element, as well as the structure of the surface sites all impact CO_2_ activation on the SAAs. Thus, it is challenging to establish simple (*e.g.*, linear) correlations describing an effective CO_2_ activation. In particular, the above discussion highlights that interesting scenarios might escape the overall (global) trends. To obtain such correlations, termed rules, we collected the 24 candidate descriptive parameters described in Table 1 for each of the 199 structures and used this information as input for the SGD AI analysis.

### 3.2 Identifying Rules Describing Large C–O Bond Elongation in SAAs

The conversion of CO_2_ into valuable products requires breaking at least one of its bonds. Hence, large values of Δ*d*_max_^C–O^ can reflect high activity of the SAA surface sites. For this reason, Δ*d*_max_^C–O^ is chosen as the target in our SGD study. The histogram showing the distribution of Δ*d*_max_^C–O^ in the data set of 199 structures (Figure 3d, in gray) highlights that the mean bond elongation is 0.103 Å. As only a few structures provide bond elongations larger than 0.15 Å, the strong activation of CO_2_ is an exceptional situation among the different SAA adsorption sites.

We used the SGD approach to identify descriptions of surface sites of SAAs presenting large values of the target Δ*d*_max_^C–O^. For this purpose, we use the normalized positive-mean-shift utility function (see Supporting Information eq S8). Table 1 shows the candidate descriptive parameters used in the SGD studies. After sectioning the φ_*i*_-space, SGD analyzed half a million SGs and identifies those with high  values. In Figure 3e, we show the 15,000 top-rated SGD solutions with respect to the quality function (eq 4). These solutions are shown in a utility-function  vs coverage plot. The SG maximizing , denoted σ_max(*Q*)_, has a Δ*d*_max_^C–O^ mean of 0.118 Å, a coverage of 0.387, and is displayed in green in Figure 3d,e. We introduce the rules associated with σ_max(*Q*)_ and the corresponding surface sites in the Supporting Information Section S3.4. This SG contains surface sites associated with larger bond elongation compared to the entire data set. Nevertheless, σ_max(*Q*)_ includes a significant fraction of systems with relatively small values of Δ*d*_max_^C–O^.

In order to identify SGs focusing on the large Δ*d*_max_^C–O^, *i.e.*, associated with more outstanding distributions of the target, we analyzed the Pareto front of SGD solutions with respect to the objectives coverage and utility function (magenta points in Figure 3e).^67^ In multiobjective optimization, a Pareto front is defined as the set of solutions for which no single objective can be improved without deteriorating at least one other objective. Thus, the solutions in the Pareto front reflect an optimal trade-off between competing objectives. This analysis allows us to take into account multiple trade-offs between the two conflicting objectives of SGD: coverage and utility. We focus on the SG with the highest utility value in the Pareto front. Denoted as σ_selected_, the coverage of this SG is equal to 0.131 and it displays a Δ*d*_max_^C–O^ mean of 0.132 Å. The target distribution in σ_selected_ is shown in blue in Figure 3d,e. This SG is more focused on the outstanding situation compared to the SG that maximizes the quality function. From the 24 offered candidate descriptive parameters, SGD identifies four parameters as key to describe the capability of the surface sites in SAAs to strongly elongate CO_2_ bonds: the electron affinity of the adsorption site (EA_site_), the Pauling electronegativity of the adsorption site (PE_site_), the generalized coordination number of the adsorption site (gen-CN), and the radius of the SA d-orbital (*r*_d-SA_). The rules associated with the SG σ_selected_ are the following
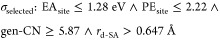
5

**Figure 4:**
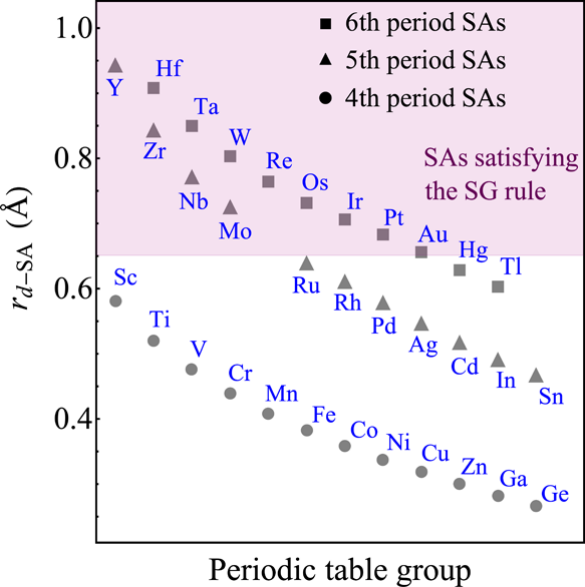
Use of the *r*_d-SA_ constraint to identify candidate SAs capable of providing a large C–O bond elongation, when embedded, in Cu, Zn, and Pd hosts. Of the 33 considered SA elements, only 13 follow the constraint for this key parameter.

EA_site_, PE_site_, and *r*_d-SA_ highlight the importance of the electronic properties of the SA and host elements for achieving CO_2_ activation. Particularly, PE characterizes the chemical interactions between adsorbates and metal surfaces.^64^ Thus, the EA_site_ and PE_site_ parameters are linked to the electronic state of the surface site and may serve as underlying properties determining the d-band center.^68^ The ability of the SAs to fine-tune the surface’s electronic properties is a fundamental factor contributing to the catalytic performance of SAAs.^34^ In particular, the rules establish that the SA should display *r*_d-SA_ > 0.647 Å. This value is larger than the hosts’ d-orbital radii equal to 0.319, 0.300, and 0.581 Å, for Cu, Zn, and Pd, respectively. The substitution of a SA induces electronic and geometric distortions in the host surface by breaking its original symmetry. This symmetry breaking can modify the adsorption properties of the surface site.^66^ Furthermore, as *r*_d-SA_ reflects the spatial distribution of the SA d-orbitals of a free atom of the SA element, this parameter describes the overlap between the surface site electronic state and the π* antibonding molecular orbitals of CO_2_. Consequently, *r*_d-SA_ might correlate with the charge transfer from the surface to the CO_2_ molecule. Figure 4 illustrates how the threshold established by the *r*_d-SA_ constraint can be used to identify SAs as potential new members of the SG associated with σ_selected_. Out of the 33 elements analyzed in Figure 4, only 13 satisfy the rule. This exemplifies the utility of the *r*_d-SA_ inequality while screening for promising SAAs for CO_2_ activation. The d-orbital radius of the elements within a given period decreases as the group number increases. Thus, there is a connection between the uncovered rules and the patterns observed in Figure 3c, where the SAs with larger d-orbital radii are also the ones favoring larger Δ*d*_max_^C–O^ (Os > Ir ≈ Pt, and Ru > Rh).

The gen-CN, in turn, denotes the importance of the geometric environment of the surface site as another key factor for CO_2_ activation. The rule on gen-CN constrains the values of this parameter to a minimum threshold. This excludes adsorption sites that present unsaturated atoms, such as the ontop and the bridge2-step sites of the (211) surfaces (see Figure S1 in the Supporting Information), and ontop and short bridge sites of the (110) surfaces. Among the surface sites not selected by the gen-CN rule, only two Os-containing SAAs exhibited a Δ*d*_max_^C–O^ larger than 0.132 Å. The selected surface sites display Δ*d*_max_^C–O^ in the range [0.057, 0.252 Å]. This implies that not only the geometry but also the SA play a fundamental role in achieving a strong CO_2_ activation. No explicit properties of the host are listed in the rules. However, EA_site_ and PE_site_ introduce a host dependence, as these quantities are averages among all the atoms that are part of the adsorption sites (SA + host). While the analysis and interpretation of the individual rules for each key parameter provides insights into the underlying properties governing CO_2_ activation on SAAs, they should always be considered collectively. In other words, only by using the full set of rules in σ_selected_ can we accurately describe large C–O bond elongations in SAAs.

The 26 surface sites that are part of σ_selected_ are presented in Table 2 and highlighted by crosses in Figure 3a–c. The selected SG contains surface sites of SAAs associated with the three considered hosts. This means that SAAs based on Cu, Zn, and Pd hosts can activate CO_2_ effectively. The geometries of the surface sites in σ_selected_ for the Cu host are bridge, hollow, and long bridge and they are present in the (111), (100), and (110) surfaces. In the case of the Zn host, ontop and bridge surface sites of the (0001) and (0001)-step surfaces are part of σ_selected_. Finally, for the Pd host, the sites in σ_selected_ are bridge, ontop, long bridge, 4-fold hollow, and fcc-s. These sites are present in the Pd surfaces (111), (100), (110), and (211). Each of these sites is displayed in the Supporting Information Figure S1. Only two SA chemical elements are contained in σ_selected_, namely Os and Ir. Overall, the analysis of the systems in σ_selected_ reveals that the electronic and geometric environment mostly provided by bridge sites with Ir and Os SAs are key for CO_2_ activation.

**Table 2:** SAAs and Surfaces Belonging to the Selected SG

host	surface	geometry of sites	SAs
	111	bridge	
Cu	100	bridge, hollow	Os
	110	long bridge	
Zn	0001	ontop,^a^ bridge	Os, Ir
	0001-step	bridge1-s, fcc-s	
Pd	111	bridge	Os, Ir
	100	ontop,^a^ bridge	
	110	long bridge, 4-fold hollow	
	211	bridge1-s, bridge3-s, fcc-s	

a Chemisorbed CO_2_ displays an η^3^ configuration.

The rules uncovered by SGD provide physical insights by identifying the key parameters favoring the effective CO_2_ activation on SAAs. In addition to the physical insights, the SG rules provide an efficient way to search for new SAA sites containing different elements or presenting different geometries. Indeed, the rules provide criteria that a surface site needs to satisfy in order to activate CO_2_. These criteria are based on basic parameters. Thus, the SG rules can identify new promising systems among large amounts of candidate materials without explicitly evaluating the CO_2_ chemisorption for the candidate materials. In particular, it is desirable to identify SAAs that can activate CO_2_ and contain more earth-abundant elements than those in the identified SG (Os and Ir). In the following section, we perform the screening of alloys considering a larger number of candidate surface sites.

### 3.3 Exploiting the SG Rules to Identify Candidate Alloys

We used the SG rules to identify SAAs in a larger *candidate space* compared to the training set. The chosen candidate space contains 20 SA chemical elements (yellow elements in Figure 2). The surface terminations, and adsorption sites considered in this analysis are shown in Table 3. We focused on bridge adsorption sites, as this site geometry is present in all of the surface terminations within σ_selected_. More specifically, three bridge sites were taken into account: the long bridge in Cu(110), and the bridge sites of Zn(0001) and Pd(100). In total, 60 surface sites are taken into account in this screening.

**Table 3:** Host Surfaces and Bridge Sites Considered in the Screening of SA and DA Alloys Suitable for CO_2_ Activation^a^

				rules’ selected sites			not selected sites	
material	host surface	studied site	candidate sites	total	tested	verified	tested	verified
	Cu(110)	long bridge	20	8	8	8	5	4
SAA	Zn(0001)	bridge	20	8	8	6	5	4
	Pd(100)	bridge	20	7	7	7	5	3
	Cu(100)	bridge	496	133	5	5	5	2
DAA	Pd(111)	bridge	496	125	5	4	5	5
	Pd(100)	bridge	496	125	5	3	5	2

a In the case of the SAAs, we used the 20 SAs highlighted in yellow in Figure 2. For DAAs, we used the set of 33 atoms highlighted in Figure 2 (green and yellow). In the case of DAAs, *r*_d-SA_ is taken as the average of the two substituted atoms. Moreover, additional DFT-mBEEF calculations confirmed the rules’ capability to predict an effective CO_2_ activation.

From the 60 SAA surface sites considered in the screening, 23 fulfill the propositions in σ_selected_. An example of how the *r*_d-SA_ constraint can be used to select promising SAs candidates can be seen in Figure 4. We performed new DFT-mBEEF calculations to confirm whether the SAAs selected by the SG rules indeed provide significant C–O bond elongation in chemisorbed CO_2_. The computational settings for these geometry optimizations were similar to the ones described in Section 2.1. We evaluated the 23 systems selected by the SG rules. These results are displayed in the upper half of Figure 3f. In this figure, the orange bins correspond to the distribution of Δ*d*_max_^C–O^ values for the 23 systems. Twenty-one of the 23 tested adsorption sites activate CO_2_. These systems contain the SA elements Y, Zr, Nb, Mo, Hf, Ta, W, and Re on the Cu(110) surface, the SA elements Y, Nb, Mo, Ta, W, and Re on the Zn(0001) surface, and the SA elements Y, Zr, Nb, Mo, Hf, Ta, and Re on the Pd(100) surface. The average Δ*d*_max_^C–O^ among these 23 sites is equal to 0.139 Å. The system displaying the largest elongation among the tested sites is Hf@Cu(110) (Δ*d*_max_^C–O^ = 0.235 Å). This large bond elongation is closer to the maximum value within the training data set, of 0.252 Å, which is associated with a long bridge in an Os@Cu(110) surface. Two sites selected by the SG rules do not activate CO_2_: Zr@Zn(0001) and Hf@Zn(0001). Additionally, we also use DFT-mBEEF to evaluate CO_2_ activation on 15 sites that were not selected by the SG rules. The distribution of Δ*d*_max_^C–O^ values corresponding to these 15 systems is shown in Figure 3f as purple bins. With a mean bond elongation of 0.04 Å, 11 of the 15 tested systems did not activate CO_2_. The largest value of Δ*d*_max_^C–O^ among these 15 systems is 0.178 Å, lower than the corresponding value among the selected SAA surface sites (0.235 Å). Overall, σ_selected_ provides good criteria to identify sites where CO_2_ can be activated in SAAs.

In addition to SAAs, we have also considered dual-atom alloys (DAAs) in our screening. DAAs are composed of a host and two different chemical elements A and B in close proximity, denoted (A, B)@host.^52,53^ DAAs have recently received attention since the synergy between the two atoms in these systems can be exploited to efficiently activate molecules, such as ethanol,^52^ and CO_2_.^53^ The design of DAAs is more challenging compared to the case of SAAs, as the number of possible combinations of elements and surface sites increases substantially. Our studies on the chemisorption of CO_2_ on SAA surface sites indicate that the SA influence on the molecule’s activation is a localized phenomenon (Supporting Information Section S1.2). As a dual-atom pair substitution provides a localized symmetry-breaking site in the host metal surface as in the case of SAAs, extending the use of the SG rules to DAAs might be possible. Hence, the SG rules could accelerate the discovery of DAA capable of activating CO_2_. For the DAA screening, we considered bridge sites of the following hosts and surface terminations Cu(100), Pd(111), and Pd(100). The 33 SA chemical elements highlighted in green and yellow in Figure 2 were included in this screening. The number of DA bridge sites that can be constructed based on these SA and host surfaces is 1488. The parameter *r*_d-SA_ in expression 5 is defined based on one SA element. Thus, to apply the constraint on *r*_d-SA_ to the screening of surface sites in DAAs, we extended the definition of *r*_d-SA_ by considering the average of the d-orbital radii of the two elements A and B in the DAA systems. The remaining parameters in expression 5 can be evaluated for DAAs using the definitions discussed previously.

By applying the SG rules, we identified 383 surface sites of DAAs likely to provide large C–O bond elongations. Then, we randomly selected 15 out of these 383 systems, five DAA sites per surface, and performed new DFT-mBEEF calculations to validate the predictions. These results are summarized in the lower half of Figure 3f. The orange bins show the distribution of Δ*d*_max_^C–O^ values corresponding to these 15 systems. Twelve of the tested bridge sites are able to activate CO_2_. These sites are, for each host surface, the following: (Cd, W), (Mo, Pd), (Sc, Hf), (Zr, Ag), (Y, Pd)@Cu(100); (Nb, Mo), (Re, Pt),(Rh, Hf),(Sc, W)@Pd(111); (Mo, Hf), (Ti, Ta),(Ta, Pt)@Pd(100). The average Δ*d*_max_^C–O^ among these 15 sites is equal to 0.111 Å. The systems displaying the largest C–O bond elongation among the tested sites are (Re, Pt)@Pd(111) and (Ta, Pt)@Pd(100), with Δ*d*_max_^C–O^ values of 0.190 and 0.187 Å, respectively. Three of the selected DAAs, (Zr, Ag)@Pd(111) and (Nb, Hg), (Y, Ag)@Pd(100), did not present CO_2_ activation. Finally, we evaluated CO_2_ activation on 15 of the DAA surface sites that were not selected by the SG rules. The purple bins of the lower panel in Figure 3f show the distribution of Δ*d*_max_^C–O^ among these 15 systems. Eight sites did not show any activation and the average Δ*d*_max_^C–O^ value across the 15 sites is 0.059 Å. Even though the SG rules missed some DAA systems providing relatively large Δ*d*_max_^C–O^ and were not as effective as they were for SAAs, they successfully identified structures providing significant Δ*d*_max_^C–O^ values among the tested DAA sites. This result is remarkable given that the rules were only trained on the much simpler SAA systems. We stress that the SGD rules can be systematically improved by retraining with more data (*e.g.*, the data related to DAAs), providing rules that better describe an effective CO_2_ activation on both SA and DA alloy systems.

In addition to the surface reactivity, the synthesizability, and stability of a catalyst during the operation are crucial design criteria in heterogeneous catalysis. Indeed, the migration of metal atoms and the segregation of different metal phases might occur in SAAs and DAAs.^69,70^ As proxies for the synthesizability and stability of the considered alloys, we evaluated the formation and segregation energies of the SAAs used for training SGD by using DFT-mBEEF calculations. The formation energy reflects the thermodynamic stability of the SAA system compared to the pure-metal phases. The segregation energy quantifies the thermodynamic preference of the SA to occupy the surface of the host atom rather than the subsurface. Our results show that the formation energies strongly depend on the host and SA. SAAs with favorable as well as unfavorable formation and segregation energies are present in our data set. Most of the SAAs in the identified SG, associated with large C–O bond elongation in chemisorbed CO_2_, are unstable. However, we observe SAAs presenting a reasonable compromise between stability and CO_2_ activation capacity, making them promising candidates for further investigation. Under operating conditions, the interaction of the SA with adsorbates in the reaction mixture can influence the alloy’s stability.^70^ Therefore, adsorbate-induced effects must be considered to obtain a deeper understanding of the SAAs’ stability under realistic reaction conditions. The discussion of our stability tests is available in Section S4 of the Supporting Information.

An active catalyst for CO_2_ hydrogenation likely displays a good capability of activating CO_2_ and H_2_. However, a selective catalyst should also favor the formation of the desired products by interacting with hydrogen and other reaction intermediates in an appropriate manner. Microkinetic and mesokinetic modeling^71,72^ are able to quantitatively link the microscopic behavior of active sites to the macroscopic performance of the material. However, such kinetic analysis requires a detailed mechanistic description of reaction pathways leading to specific products, such as methanol and ethanol. Because the results of our SGD study can significantly narrow down the pool of SA and DAAs capable of achieving CO_2_ activation, the first critical step in the molecule’s conversion, they guide subsequent, detailed mechanistic and kinetic investigations on promising systems for specific CO_2_ conversion processes. Besides, the SGD analysis can be extended to investigate other critical steps in the CO_2_ conversion pathways leading to specific products. For instance, the stability of formate intermediate in the molecule’s hydrogenation to methanol has been proposed to be crucial.^11,39^ A detailed analysis of Pd-based SAAs for CO_2_ hydrogenation to methanol based on DFT-mBEEF calculations will be discussed in an upcoming contribution.

Finally, we note that the reaction conditions were not taken into account in this work. Nonetheless, if the applied reaction conditions (*e.g.*, temperature, pressure) are harsh, the surface of the catalyst might restructure. Such restructuring is typically unknown. Previously, the blending of high-quality theoretical and experimental data has shown that AI can identify correlations that take into account the experimental reaction conditions.^73,74^ These approaches enabled the design of new materials. Thus, the incorporation of experimental data into SGD studies describing CO_2_ conversion on SA and DA alloys is a promising route for addressing the impact of reaction conditions on the catalytic performance. This will require, however, systematic and rigorous experimental procedures^75^ for the characterization and the evaluation of stability and reactivity of series of SAAs and DAAs.

## 4 Conclusions

In this work, we used high-quality data generated by DFT-mBEEF calculations and applied the SGD AI approach to study the CO_2_ activation in 36 SAAs based on Cu, Zn, and Pd hosts. From 24 basic candidate descriptive parameters, SGD selected four parameters as key to characterize SAA surface sites capable of strongly elongating the C–O bonds of the chemisorbed molecule. These key properties highlight the importance of the SA nature (radius of its d-orbitals) and the surface site’s electronic (the electron affinity and Pauling electronegativity of the surface site) and geometric properties in achieving an effective CO_2_ activation. The rules provided by SGD connect these key parameters to the desired bond elongation and can be used for materials design. Indeed, from a total of 1548 candidate systems, the rules enabled a fast screening and prediction of SA and DA alloys of new potential members of the outstanding SG. Through new DFT-mBEEF simulations, we tested and confirmed the rules’ capability to correctly predict the strong CO_2_ activation on SA and DA alloys beyond the chemical space used to train SGD. We hope this work will encourage the use of high-quality data with focused AI approaches, like SGD, to accelerate the design of materials for catalysis. Crucially, having access to experimental data capturing phenomena that are hard to include in calculations, such as surface reconstruction and mass/energy transport, will boost the capabilities of data-centric AI approaches.

## Data Availability

A GitHub repository with additional details on the alloy screening, scripts for running SGD, and a Jupyter notebook to reproduce the results discussed in this work, is available at https://github.com/HerzainR/CO2-Activation-in-SAAs. The input and output files for the DFT calculations are available in the NOMAD repository at http://doi.org/10.17172/NOMAD/2024.05.10-1.

## Supporting Information Available

Electronic Supporting Information with additional details on the DFT-mBEEF calculations, the use of SGD, and the stability tests of the SAAs considered in this study (PDF)
The data set containing the candidate descriptive parameters for the 199 chemisorbed structures is available through the co2_activation_in_saa.xlsx (XLSX)
