# Supplementary material for: Rules Describing CO2 Activation on Single-Atom Alloys from DFT-Meta-GGA Calculations and Artificial Intelligence
Source: ACS Catal. 2025 Feb 4;15(4):2916–26. doi: 10.1021/acscatal.4c07178 (PMC11851785; doi:10.1021/acscatal.4c07178)
Supplement: Supplementary file 1 — cs4c07178_si_001.pdf [file cs4c07178_si_001.pdf]

Electronic Supporting Information (ESI)

Rules Describing CO_2 Activation on Single-Atom Alloys from DFT-meta-GGA Calculations and Artificial Intelligence

Herzain I. Rivera-Arrieta* and Lucas Foppa*

The NOMAD Laboratory at the Fritz Haber Institute of the Max Planck Society, Faradayweg 4-6, D-14195, Berlin, Germany

Correspondence: rarrieta@fhi-berlin.mpg.de; foppa@fhi-berlin.mpg.de

S1 DFT Calculations

In this Section, we provide details on the DFT-mBEEF calculations utilized to evaluate the CO_2 interaction with the surfaces of single-atom alloys (SAAs). All the calculations were performed with the FHI-aims package.[1]

S1.1 Exchange-Correlation Functional

The performance of different exchange-correlation functionals for Cu, Zn, and Pd, was previously benchmarked against their experimental bulk properties and surface energies.[2] In these studies, the mBEEF[3] functional provides the lowest errors (on average) for these three metals. When benchmarked against the random-phase approximation for adsorption energies of atoms and molecules on metal surfaces, mBEEF shows a mean signed error (MSE) of −0.05 eV.[4] This MSE for mBEEF is lower than the MSEs observed for PBE and RPBE (−0.09 and 0.12 eV, respectively). However, experimental vibrational frequencies of adsorbed ethylene and acetylene on Cu, Rh, Pd, and Pt surfaces have been used to assess the accuracy of RPBE and mBEEF functionals.[5] With mean percentage errors of 0.64% and -3.88%, respectively, RPBE performs better than mBEEF. Nonetheless, the difference is small enough to consider both functionals as good choices for studying molecular adsorptions on metals.

In order to evaluate the influence of the exchange-correlation functional on the description of CO_2 adsorption on SAAs, we compared the results for three exchange-correlation functionals: PBE, RPBE, and mBEEF. For each functional, we started by optimizing the (bulk) unit cells of Cu, Zn, and Pd host elements. Then, we model the CO_2 adsorption on three slabs (see Section 1.2 for the slab details): Ru@Cu(111), Rh@Zn(0001), and Os@Pd(111). The simulations used the FHI-aims "light" basis set. We were interested in 3 main parameters, namely, the molecule's adsorption energy ($E_{\text{ads}}^{\text{CO}_2}$), the elongations of the C−O bonds ($\Delta d_i^{\text{C-O}}$), and the OCO angle. We evaluate $E_{\text{ads}}^{\text{CO}_2}$ according to Eq. 1 of the main text. The expression for evaluating the bond elongations is

$$\Delta d_i^{\text{C-O}} = \text{d}_{i,\,\text{chem}}^{\text{C-O}} - \text{d}_{\text{equil}}^{\text{C-O}}. \tag{S1}$$

Here, $\text{d}_{i,\,\text{chem}}^{\text{C-O}}$ is one of the two C−O distances in the chemisorbed CO_2, and $\text{d}_{\text{equil}}^{\text{C-O}}$ refers to the distance between C and O in an isolated CO_2 molecule.

Table S1 shows the results for the PBE, RPBE, and mBEEF functionals. The geometrical parameters are comparable among the three functionals. For instance, the maximum deviation is 0.012 Å among $\Delta d^{\text{C-O}}$ values, and 2 degrees for the OCO angle. These results show how the selected target for our Artificial Intelligence studies, the C−O bonded elongation, is not strongly affected by the exchange-correlation functional. In the case of the adsorption energies, RPBE shows differences up to 0.41 and 0.32 eV against the results provided by mBEEF and PBE, respectively. Moreover, when compared against PBE and mBEEF functionals, RPBE tends to predict weaker CO_2 adsorption energies. These results are consistent with the general trends observed by Schmidt et al.[4] The calculations in the following Subsections refer only to DFT-mBEEF calculations.

Table S1: Optimized parameters for a chemisorbed CO_2 molecule on SAAs obtained from mBEEF, PBE, and RPBE density functionals. We employed 3×3×7 slab models and the FHI-aims "light" basis set. For simplicity, the figures in the table only display two layers.

Ru@Cu(111)

Functional	$E_{ads}^{CO_2}$ (eV)	Δd_1^{C-O} (Å)	Δd_2^{C-O} (Å)	$\measuredangle$ OCO
mBEEF	−0.337	0.067	0.083	138.58
PBE	−0.218	0.062	0.078	140.06
RPBE	0.084	0.058	0.080	140.10

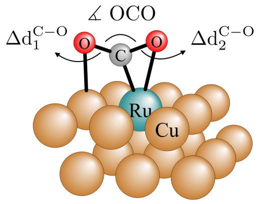

Rh@Zn(0001)

Functional	$E_{ads}^{CO_2}$ (eV)	Δd_1^{C-O} (Å)	Δd_2^{C-O} (Å)	$\measuredangle$ OCO
mBEEF	0.175	0.103	0.103	125.13
PBE	0.271	0.096	0.096	126.85
RPBE	0.599	0.095	0.095	127.13

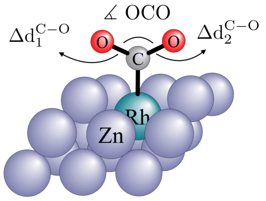

Os@Pd(111)

Functional	$E_{ads}^{CO_2}$ (eV)	Δd_1^{C-O} (Å)	Δd_2^{C-O} (Å)	$\measuredangle$ OCO
mBEEF	−0.238	0.142	0.035	130.16
PBE	−0.198	0.130	0.030	131.62
RPBE	0.138	0.130	0.031	131.63

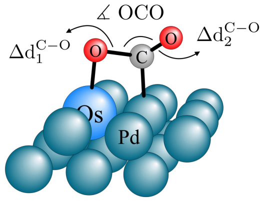

S1.2 Atomistic Models

As the starting point, we optimize the (bulk) unit cells of Cu, Zn, and Pd host elements. For Cu and Pd, which present the face-centered-cubic (FCC) structure, we studied the (111), (110), (100), and (211) surface terminations. For Zn, which exhibits the hexagonal-closed-packed (HCP) structure, we considered the (0001) and (0001)-stepped surfaces. To model the (111), (100), (110), and (0001) surfaces, we used slabs with (3×3×7) dimensions. For the (211) and (0001)-stepped surfaces, the slab dimensions are (6×3×7). For each slab, the lower three layers remained fixed at the host's bulk configuration, and the atoms in the four upper layers were allowed to relax.

To avoid the presence of any potential dipole field in our non-symmetric slab models, we include the dipole correction provided by FHI-aims. One adsorbate molecule per slab is considered. Thus, the CO_2 coverage for the flat and step surfaces are 1/9 and 1/18 monolayers, respectively. As the starting point of all the simulations, we placed the CO_2 molecule $\sim$ 1.8 Å above, and in a parallel orientation, to each one of the surface adsorption sites. In Fig. S1, we show the 24 considered surface sites on the (111), (100), (110), and (211) in the FCC hosts Cu and Pd, and the 17 sites for the (0001) and (0001)-stepped surfaces in HCP Zn. The total number of sites for the monometallic systems is 41, and, if we consider 12 SAAs per host metal, the total number of surface sites is 780.

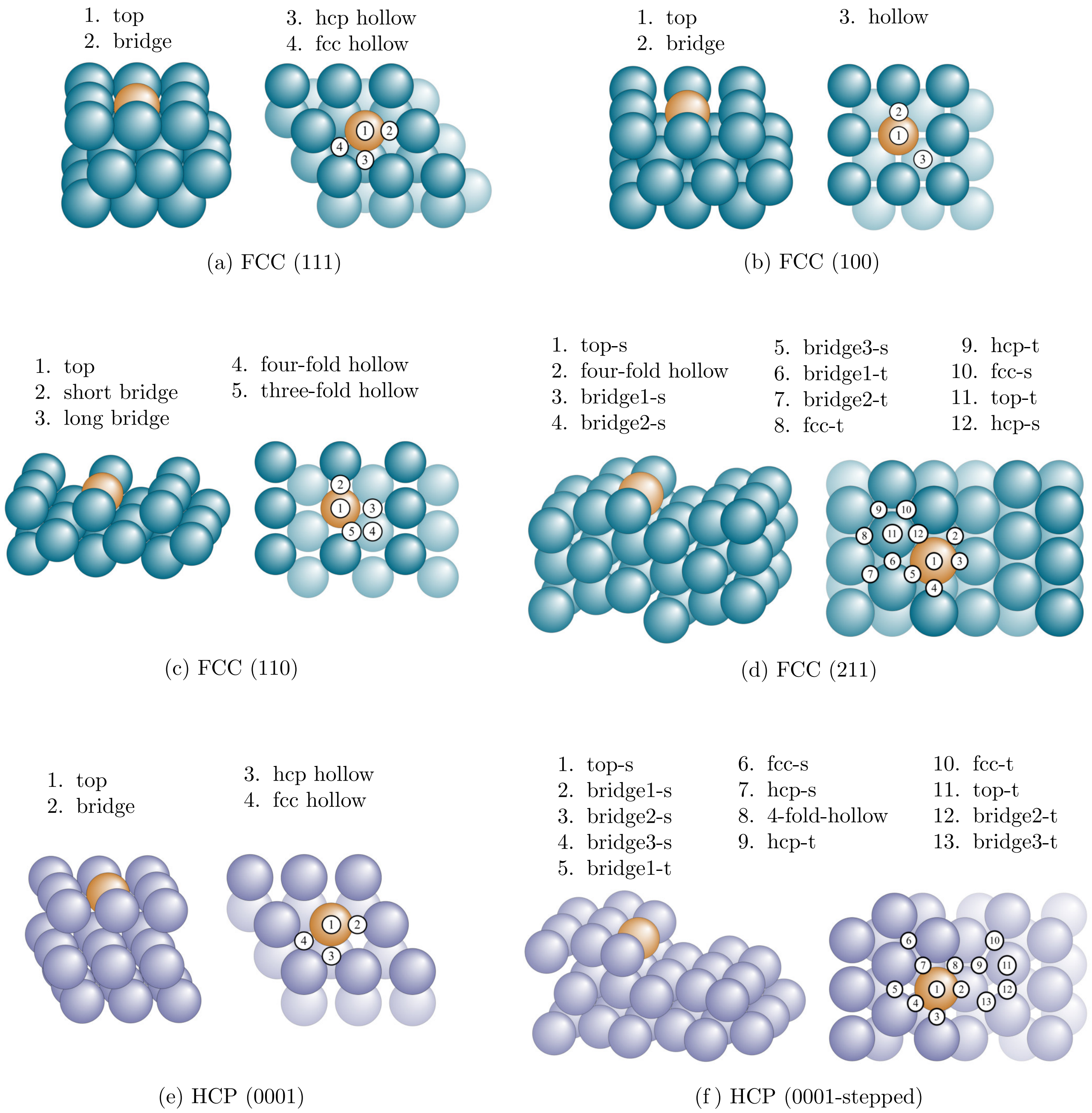

Fig. S1: Surface adsorption sites in the SAAs studied in this work (FCC and HCP). The sites are defined so they remain as close as possible to the single atom (SA). The blue and purple spheres represent the host metals in the FCC and HCP surfaces. In both structures, FCC and HCP, the dark orange sphere corresponds to the guest SA. Although all the surfaces considered in our work have 7 layers, this figure only displays 3 of them for simplicity.

In addition to the surface sites composed among the SA and host atoms, additional surface sites are available for CO_2 adsorption in SAAs. These sites might be in the vicinity or even far away from the SA. To understand whether the properties of the surface sites in the vicinity of the SA are comparable to those on monometallic systems, we evaluated the CO_2 interaction with those different sites by considering the bridge and long bridge sites in Pd(100) and Pd(110), respectively. In Table S2, we compare the $E_{\text{ads}}^{\text{CO}_2}$, the maximum C−O elongation ($\Delta d_{\text{max}}^{\text{C−O}}$), and the OCO angle, among several CO_2 chemisorbed structures. We notice negligible differences in

the geometric parameters between adsorption sites in the vicinity of the SA and in the corresponding site of the monometallic system. The $E_{ads}^{CO_2}$ differences are more notorious, but small enough (< 0.04 eV) to say that the SA influence is very local. Therefore, we only included the adsorption on the monometallic systems in our analysis.

Table S2: The influence of the single-atom (SA) in the geometry and adsorption energy when CO_2 is chemisorbed in a surface site without direct interaction with the SA is small. We do not observe significant geometry or energy differences between the CO_2 chemisorption on sites composed only by host elements in the SAA surfaces and equivalent sites on monometallic surfaces ($\Delta E_{ads}^{CO_2} < 0.04$ eV). These simulations used 3×3×7 slab models and the FHI-aims "light" basis set. The figures in the table only display two layers for simplicity.

Surface	$E_{ads}^{CO_2}$ (eV)	ΔE (eV)	Δd_{max}^{C-O} (Å)	$\measuredangle$ OCO
Pd(100)	−0.091	-	0.085	138.78
Cu@Pd(100)	−0.113	0.022	0.085	138.66
Zn@Pd(100)	−0.103	0.012	0.083	139.49
Pt@Pd(100)	−0.060	−0.031	0.085	138.62

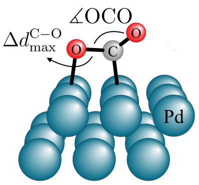

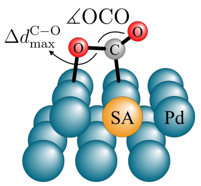

Surface	$E_{ads}^{CO_2}$ (eV)	ΔE (eV)	Δd_{max}^{C-O} (Å)	$\measuredangle$ OCO
Pd(110)	−0.178	-	0.086	135.30
Cu@Pd(110)	−0.184	0.006	0.084	135.70
Zn@Pd(110)	−0.175	−0.003	0.083	135.95
Pt@Pd(110)	−0.136	−0.042	0.083	135.98

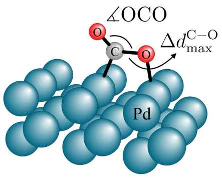

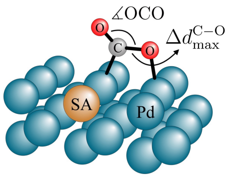

S1.3 Basis Sets and K-Grid Density

The FHI-aims code[1] is based on numerical atom-centered orbitals and with several choices for the basis sets, including the standard "light", "intermediate", "tight", and "very tight" settings. These basis sets control the number of radial functions considered in the calculation and the grid used during the numerical integration of the Hamiltonian matrix elements. In order to assess the influence of the choice of basis set on our results, we evaluated CO_2 chemisorption on 3 SAAs by considering the standard settings "light", "intermediate", and "tight". We evaluated the root mean square error (RMSE) in the atomic positions with respect to the structure optimized with "tight" settings. We also compared the $E_{ads}^{CO_2}$ values obtained with the three basis sets. These results are summarized in table S3. The maximum RMSE for the atomic positions is 0.150 Å. This analysis shows that the geometries have a very modest dependence on the basis set for the considered systems. Thus, all the geometry optimizations employed "light" settings. Nonetheless, we observe differences up to 0.11 eV for the $E_{ads}^{CO_2}$ between "light" and "tight" settings. As the energy differences in these weakly bound systems can be relevant to our study, we analyze the use of different basis sets in more detail.

Table S3: Geometry optimization of one CO_2 molecule on 3×3×7 SAAs slabs using three different FHI-aims basis sets. While the atomic positions seem to be rather independent of the basis, $E_{ads}^{CO_2}$ is more sensitive to this choice.

FHI-aims	Atomic positions RMSE (Å)			$E_{ads}^{CO_2}$ (eV)		
basis set	Ru@Cu(111)	Rh@Zn(0001)	Os@Pd(111)	Ru@Cu(111)	Rh@Zn(0001)	Os@Pd(111)
Tight	0	0	0	−0.399	0.070	−0.357
Intermediate	0.028	0.007	0.026	−0.260	0.190	−0.208
Light	0.040	0.150	0.128	−0.316	0.113	−0.238

To analyze the adsorption energy convergence with respect to the basis set and the **k**-grid density, we evaluated the CO_2 chemisorption on a 3×3×7 Ru@Cu(111), Rh@Zn(0001), and Os@Pd(111) slab model surfaces considering two sets of simulations. First, for each basis set, we performed the geometry relaxation of each system. In each one of the tested SAAs surfaces, the $E_{ads}^{CO_2}$ converges when the **k**-grid density is below one **k**-point per $(0.018 \times 2\pi)$ Å^{-1}. The second set of tests involved single-point calculations on top of the optimized structures using the "light" basis set and a **k**-point density below $(0.018 \times 2\pi)$ Å^{-1}. Adsorption energy differences up to 0.12 eV are evident between "light" and "tight" basis sets.

Based on these results, we use the following two-step procedure for all our DFT-mBEEF simulations:

a) As the basis does not significantly affect the geometric parameters of the optimized structures, all the geometry optimizations used the "light" basis set and a **k**-grid density below one **k**-point per $(0.018 \times 2\pi)$ Å^{-1}.

b) To assure a converged value for the $E_{ads}^{CO_2}$, an additional single-point calculation with "tight" settings was performed on the structures where CO_2 chemisorbs.

Table S4: Adsorption energy dependence on the **k**-point density and the FHI-aims basis set. These results correspond to the CO_2 chemisorption on a 3×3×7 Ru@Cu(111) slab. The reference structure for the single-point calculations is the one optimized with a **k**-grid density equal to $(0.019 \times 2\pi)$ Å^{-1}.

**k**-point ×	$E_{ads}^{CO_2}$ (eV)^a				$E_{ads}^{CO_2}$ (eV)^b			
2 π (Å^{-1})	Light	Intermediate	Tight	Really tight	Light	Intermediate	Tight	Really tight
0.0507	−0.285	−0.237	−0.377	−0.377	−0.274	−0.214	−0.354	−0.356
0.0253	−0.310	−0.259	−0.399	−0.399	−0.315	−0.250	−0.394	−0.394
0.0190	−0.332	−0.281	−0.421	−0.421	−0.336	−0.271	−0.412	−0.416
0.0152	−0.315	−0.260	−0.399	−0.400	−0.319	−0.249	−0.394	−0.394
0.0126	−0.314	−0.261	−0.401	−0.401	−0.318	−0.247	−0.384	−0.395
0.0108	−0.319	−0.266	−0.396	−0.396	−0.310	−0.243	−0.388	−0.389
0.0076	−0.315	−0.261	−0.400	−0.396	−0.311	−0.249	−0.393	−0.390

[a] Values from geometry optimizations. [b] Values from single point calculations.

Table S5: Adsorption energy dependence on the **k**-point density and the FHI-aims basis set. These results correspond to the CO_2 chemisorption on a 3×3×7 Rh@Zn(0001) slab. The reference structure for the single-point calculations is the one optimized with a **k**-grid density equal to $(0.0184 \times 2\pi)$ Å^{-1}.

**k**-point ×	$E_{ads}^{CO_2}$ (eV)^a				$E_{ads}^{CO_2}$ (eV)^b			
2 π (Å^{-1})	Light	Intermediate	Tight	Really tight	Light	Intermediate	Tight	Really tight
0.0491	−0.022	0.026	−0.090	−0.089	0.236	0.327	0.199	0.199
0.0245	0.064	0.143	0.024	0.024	0.109	0.204	0.076	0.076
0.0184	0.177	0.242	0.123	0.123	0.180	0.274	0.147	0.147
0.0147	0.112	0.189	0.068	0.068	0.125	0.220	0.093	0.092
0.0122	0.120	0.194	0.077	0.075	0.126	0.221	0.094	0.093
0.0105	0.139	0.208	0.090	0.089	0.143	0.238	0.111	0.111
0.0073	0.122	0.199	0.080	0.081	0.135	0.230	0.102	0.102

[a] Values from geometry optimizations. [b] Values from single point calculations.

Table S6: Adsorption energy dependence on the **k**-point density and the FHI-aims basis set. These results correspond to the CO_2 chemisorption on a 3×3×7 Os@Pd(111) slab. As reference structure for the single-point calculations, we used the one obtained with a **k**-grid density equal to $(0.0175 \times 2\pi)$ Å^{-1}.

**k**-point ×	$E_{\mathrm{ads}}^{\mathrm{CO_2}}$ (eV)[a]			$E_{\mathrm{ads}}^{\mathrm{CO_2}}$ (eV)[b]			
2 π (Å^{-1})	Light	Intermediate	Tight	Light	Intermediate	Tight	Really tight
0.0466	−0.243	−0.214	−0.362	−0.238	−0.182	−0.331	−0.329
0.0233	−0.236	−0.207	−0.356	−0.236	−0.178	−0.329	−0.328
0.0175	−0.238	−0.209	−0.358	−0.239	−0.181	−0.332	−0.332
0.0140	−0.231	−0.202	−0.352	−0.230	−0.173	−0.323	−0.323
0.0116	−0.235	−0.206	−0.356	−0.235	−0.179	−0.329	−0.330
0.0100	−0.235	−0.206	−0.355	−0.235	−0.178	−0.328	−0.330
0.0070	−0.234	−0.206	−0.356	−0.235	−0.178	−0.328	−0.328

[a] Values from geometry optimizations. [b] Values from single point calculations.

S1.4 Spin treatment

The vast majority of the elements considered in our study are nonmagnetic. Nonetheless, the proper treatment of Co and Ni requires spin-polarized calculations as they exhibit non-zero magnetic moments. In Table S7, we show the differences between CO_2 adsorption energies ($\Delta E_{\mathrm{ads}}^{\mathrm{magnetic}}$) evaluated through spin-polarized ($E_{\mathrm{ads}}^{\mathrm{spin\text{-}collinear}}$) and spin-free calculations ($E_{\mathrm{ads}}^{\mathrm{spin\text{-}free}}$).

$$\Delta E_{\mathrm{ads}}^{\mathrm{magnetic}} = E_{\mathrm{ads}}^{\mathrm{spin\text{-}collinear}} - E_{\mathrm{ads}}^{\mathrm{spin\text{-}free}}. \tag{S2}$$

Indeed, we notice how the inclusion of the spin treatment can significantly affect $\Delta E_{\mathrm{ads}}^{\mathrm{magnetic}}$, with differences up to 0.82 eV, among several SAA surfaces with Co and Ni. Therefore, we included the spin treatment for all the SAAs containing these elements.

Table S7: Adsorption energy differences related to the magnetic character in different SAAs surfaces containing Co and Ni. These values were obtained employing the FHI-aims "light" basis set. These values do not provide an extensive list of the structures containing Co and Ni within our data set.

Surface	Site	$\Delta E_{\mathrm{ads}}^{\mathrm{magnetic}}$ (eV)	Surface	Site	$\Delta E_{\mathrm{ads}}^{\mathrm{magnetic}}$ (eV)
Co@Cu(111)	bridge	0.430	Co@Pd(111)	bridge	0.307
Ni@Cu(111)	bridge	0.001†	Ni@Pd(111)	bridge	0.185
Co@Cu(100)	hollow	0.820	Co@Pd(100)	bridge	0.388
Ni@Cu(100)	ontop	~ 0†	Ni@Pd(100)	bridge	−0.118
Co@Zn(0001)	bridge	0.334	Co@Pd(110)	Long bridge	−0.044
Ni@Zn(0001)	ontop	~ 0†	Ni@Pd(211)	bridge3-s	0.244

† Collinear optimization provided a near zero spin moment

S1.5 Non-self consistent (NSC) Hartree-potential correction (HPC) to the Hellmann-Feynman forces

In FHI-aims, the total energy evaluation at the μ iteration ($E_T^{(\mu)}$) within a self-consistent field (SCF) cycle is performed according to the Harris Functional:[1]

$$E_T^{(\mu)} = \sum_l f_l^{(\mu)} \epsilon_l^{(\mu)} - \int d\boldsymbol{r} \left[\rho^{(\mu-1)}(\boldsymbol{r}) v_{xc}[\rho^{(\mu-1)}](\boldsymbol{r})\right] + E_{xc}[\rho^{(\mu-1)}] - \frac{1}{2}\int d\boldsymbol{r} \left[\rho^{(\mu-1)}(\boldsymbol{r}) v_H^{(\mu-1)}(\boldsymbol{r})\right] + E_{NN}. \quad \text{(S3)}$$

In this expression, $\sum_l f_l^{(\mu)} \epsilon_l^{(\mu)}$ are the single-particle eigenvalues for the Kohn-Sham electronic Hamiltonian, $\rho^{(\mu-1)}$ refers to the damped density (*e.g.* a density mixed through a Pulay algorithm) of the $\mu - 1$ iteration, v_{xc} and E_{xc} are the exchange-correlation potential and energy, respectively, v_H is the Hartree potential, and E_{NN} is the internuclear repulsion. If we consider a small variation in $E_T^{(\mu)}$ due to a change in the nuclei positions ($\boldsymbol{R}_{at}$), we can derive the following expression for the forces experienced by the atoms in the system:

$$-\frac{\delta E_T^{(\mu)}}{\delta \boldsymbol{R}_{at}} = + \int d\boldsymbol{r} \left[\left(\rho^{(\mu-1)}(\boldsymbol{r}) - \rho_{KS}^{(\mu)}(\boldsymbol{r})\right) \frac{\delta v_{xc}[\rho^{(\mu-1)}](\boldsymbol{r})}{\delta \boldsymbol{R}_{at}}\right] + \int d\boldsymbol{r} \left[\left(\rho^{(\mu-1)}(\boldsymbol{r}) - \rho_{KS}^{(\mu)}(\boldsymbol{r})\right) \frac{\delta v_H^{(\mu-1)}(\boldsymbol{r})}{\delta \boldsymbol{R}_{at}}\right]$$

$$- \int d\boldsymbol{r} \left[\rho_{KS}^{(\mu)}(\boldsymbol{r}) \frac{\delta v_{ext}(\boldsymbol{r})}{\delta \boldsymbol{R}_{at}}\right] - \frac{\delta E_{NN}}{\delta \boldsymbol{R}_{at}} = + F_{at}^{v_{xc},\mathrm{NSC}} + F_{at}^{v_H,\mathrm{NSC}} - F_{at}^{\mathrm{HF}}. \quad \text{(S4)}$$

In eq. S4, $\rho_{KS}^{(\mu)}$ is the density of the Kohn-Sham orbitals for the μ step, *i.e.*, this is an unmixed density. Additionally, we define the first two terms on the right-hand side (rhs) of the equation, $F_{at}^{v_{xc},\mathrm{NSC}}$ and $F_{at}^{v_H,\mathrm{NSC}}$, as the exchange-correlation and the Hartree potential force "correction" terms, respectively. Finally, the last term on the rhs, F_{at}^{HF}, condenses the Hellmann-Feynman force terms discussed by Blum et al.[1] Only when the density achieves full self-consistency ($\rho^{(\mu-1)} = \rho_{KS}^{(\mu)}$), $F_{at}^{v_{xc},\mathrm{NSC}}$ and $F_{at}^{v_H,\mathrm{NSC}}$ are equal to zero. Thus, when these correction terms are omitted, high self-consistency in the electron density is required before the forces can be trustworthy.[6] Due to the absolute magnitude difference between v_{xc} and v_H, $F_{at}^{v_H,\mathrm{NSC}}$, is the most relevant correction term for the forces when $\rho^{(\mu-1)}$ is not yet self-consistent.

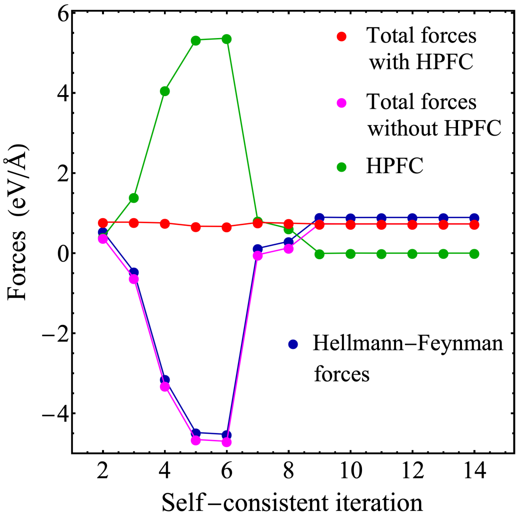

Fig. S2: Forces convergence with respect to the number of iterations in a H_2 single self-consistent cycle (d_{H-H} = 0.73 Å). This calculation used PBE as xc-functional and the "light" basis set.

In order to include $F_{at}^{v_H,\mathrm{NSC}}$ evaluation within FHI-aims force computations, a module taking care of evaluating this correction term was implemented. The module has been available since FHI-aims version 210226, and the HPC evaluation is now a default in the code. To exemplify how the HPC improves the forces' stability, let us consider a DFT-PBE calculation for a H_2 molecule where the H−H distance (d_{H-H}) is not optimized. Figure S2 plots the magnitude of the forces along the H_2 bond axis, at each iteration during a single self-consistent cycle. For simplicity, we did not plot the Pulay and the multipole forces.

When $F_{at}^{v_H,\mathrm{NSC}}$ is omitted, the initial iterations show strong fluctuations in the total forces, as expected by eq. S4. However, the addition of $F_{at}^{v_H,\mathrm{NSC}}$ leads to the stabilization of the total forces along the entire SCF cycle. As the number of SC iterations increases (*i.e.* for better-converged electron densities) the HPC approaches zero as expected. Thus, the HPC allows the use of reasonable loose thresholds for the electron density convergence without compromising the accuracy of the forces. This can also reduce the computational resources required in any FHI-aims force calculation.

We performed a benchmark to quantify the best way to exploit the HPC in our studies of the CO_2 interacting with SAAs. In particular, we aim to find an appropriate electron density convergence threshold that allows us to save computational resources, and at the same time, does not compromise the quality of the obtained geometry. Table S8 shows four independent DFT-mBEEF geometry optimizations of a single CO_2 molecule on a 3×3×7

Os@Pd(111) slab. FHI-aims default accuracy threshold ($10^{-6} \times \sqrt{\#atoms/6}\ e\, a_0^{-3}$) for the electron density provides a safe, well-converged value. Thus, the default is taken as the reference. Each structure is considered converged when the forces in the atoms reach 0.01 eV $Å^{-1}$. These calculations used the FHI-aims "light" basis set and six compute nodes of the Max Planck Computing and Data Facility cluster Raven. Each node has Intel Xeon IceLake-SP processors with 72 cores and 256 GB RAM.

Thanks to the HPC, there are no significant differences among the structures optimized through the default, $10^{-4}\, e\, a_0^{-3}$, and $10^{-3}\, e\, a_0^{-3}$ electron density thresholds. The RMSE for the geometries is lower than 0.003 Å, and their energy difference is below 0.015 meV. If the electron density threshold is equal to $10^{-3}\, e\, a_0^{-3}$ and the HPC is omitted, the optimization does not converge before the time wall (420 min). Compared with the FHI-aims convergence defaults, the calculations with the HPC provide negligible differences even if the electron density is loosely converged. Taking into account the nearly 30 % reduction in CPU time for the $10^{-3}\, e\, a_0^{-3}$ electron density threshold, we chose this value to run our geometry optimizations.

Table S8: HPC benchmark for the optimization a single CO_2 molecule on a 3×3×7 Os@Pd(111) slab. Here, we can see how the inclusion of HPC allows the obtention of stable results despite the use of loose convergence criteria for ρ.

ρ accuracy ($\times\, e\, a_0^{-3}$)	HPC active	CPU time (min)	Atomic positions RMSE (Å)	Energy diff. (meV)
2.65E-5[a]	Yes	363	0	0
10^{-4}	Yes	311	0.001	0.04
10^{-3}	Yes	265	0.003	0.13
10^{-3}	No	420[b]	–	–

[a] Default value assigned by FHI-aims.
[b] Optimization without HPC did not reach convergence in the selected time wall.

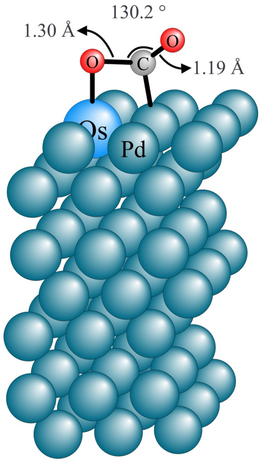

The previous discussion for the HPC only applies to the optimization of atomic positions. At the moment, no equivalent term has been added to the stress computations in FHI-aims. Thus, we recommend always using the default convergence thresholds when optimizing lattice vectors.

S2 Additional Details on the Chemisorption of CO_2 in the SAAs surfaces

S2.1 Definition of the Surface Sites and Candidate Descriptive Parameters

The candidate descriptive parameters used in the SGD analysis (see Table I in the main text) correspond to four types: host, SA, site, and site + first neighbors. The evaluation of the parameters of type "site" considers all the atoms in the surface sites where CO_2 is chemisorbed. The parameters of type "site + first neighbors" are evaluated considering, in addition to the atoms of the surface site, the neighbors of these atoms. Thus, to calculate these two types of parameters, the atoms that are part of an adsorption site need to be defined.

We determined the number of atoms in a given surface site by considering a cutoff distance (d_{cutoff}) between the surface atoms and the atoms of the chemisorbed CO_2 molecule. If a surface atom is below d_{cutoff}, this atom is considered to be part of a surface site. This cutoff distance is equal to 2.6 Å. The count of the first neighbors of the surface sites considers the closest interatomic distance of the atoms in the bulk, *i.e.*, it depends on the host element and structure (see Table S9).

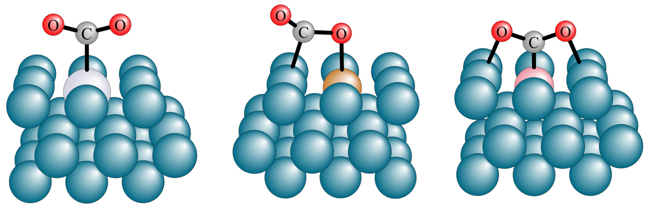

(a) η^1-CO_2 (b) η^2-CO_2 (c) η^3-CO_2

Fig. S3: (a) The molecule binds with the surface only through the C-atom. (b) A chemisorption through one of the O atoms and the C leads to an asymmetric elongation of the adsorbate bonds. (c) The three atoms in CO_2 bind the surface.

The definition of surface sites based on their distance to the CO_2 molecule is important because one single surface motif can result in different chemisorption modes of CO_2. For instance, Figure S3 shows three adsorption modes for an on-top site, termed η^1-CO_2, η^2-CO_2, and η^3-CO_2, according to the number of chemical bonds between the chemisorbed CO_2 molecule and the surface, respectively one, two, and three. In the case of η^1-CO_2 adsorption, only one surface atom is considered part of the site, denoted $\mathrm{site_{no}} = 1$. In the case of η^2-CO_2 and η^3-CO_2, two and three surface sites, respectively, are considered part of the site. Another example is shown in Figure S4. This figure shows an η^3 adsorption of CO_2 on an Os@Cu(100) hollow site. In this case, $\mathrm{site_{no}} = 4$.

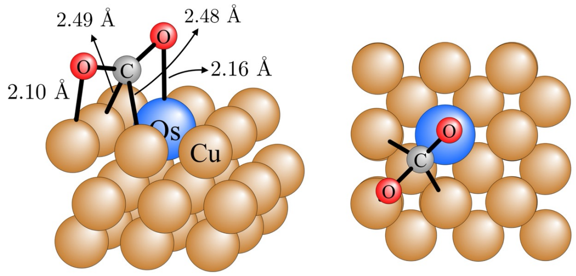

Fig. S4: η^3-CO_2 chemisorption on a hollow site in Os@Cu(100).

Table S9: Cutoff distance ($\mathrm{d_{cutoff}}$) used to count the number of atoms in the adsorption sites. In the three cases, we compare $\mathrm{d_{cutoff}}$ against the distance between neighbor atoms in the host bulk ($\mathrm{Bulk_{nnd}}$).

Host metal	$\mathrm{Bulk_{nnd}}$ (Å)	$\mathrm{d_{cutoff}}$ (Å)
Cu	2.53	2.60
Zn	2.61	2.60
Pd	2.75	2.60

S2.2 Analysis of Symmetric And Asymmetric CO_2 Chemisorption Modes

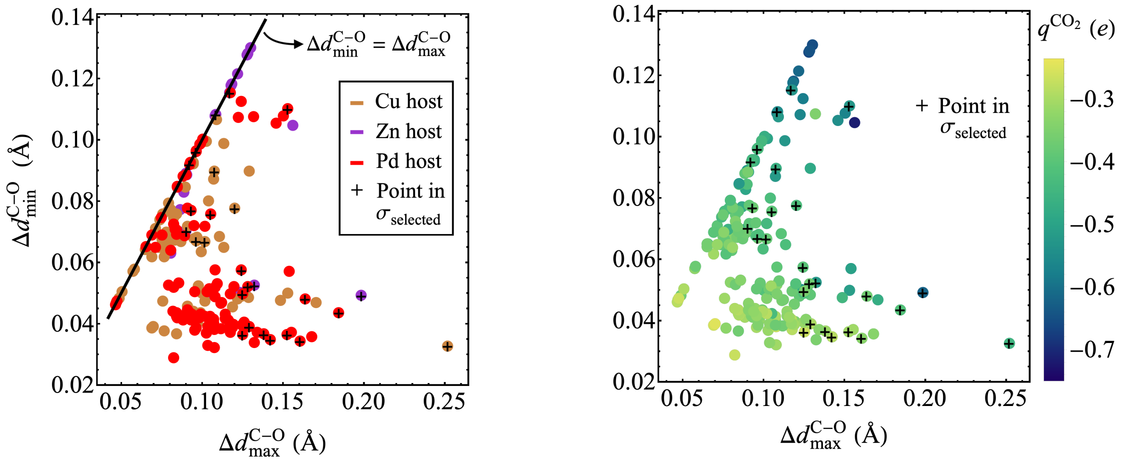

(a) (b)

Fig. S5: Plot of both C−O bond elongations after the CO_2 chemisorption on SAAs. We identify the largest elongation value as Δd^{C-O}_{max}, and the shortest as Δd^{C-O}_{min}. Among the structures in our data set, CO_2 chemisorption is either symmetric ($\Delta d^{C-O}_{min} = \Delta d^{C-O}_{max}$) or asymmetric ($\Delta d^{C-O}_{min} < \Delta d^{C-O}_{max}$). (a) The elongation values with respect to the host metals do not display any clear tendency. Nonetheless, the SG identified by the rules ($\sigma_{selected}$) shows a preference for the asymmetric chemisorption mode. (b) The total Mulliken charges on chemisorbed CO_2 (q^{CO_2}) show how a large charge transfer from the SAA surfaces is associated with larger bond elongations.

In the main text, we focused on large C−O bond elongations in chemisorbed CO_2 (see definitions in Eq. 2), and we used these values as the target for the SGD analysis. Here, we analyze the relationship between the two bond elongations in the chemisorbed CO_2 molecule. We denote the largest and the shortest bond elongations as $\Delta d^{\mathrm{C-O}}_{\mathrm{max}}$ and $\Delta d^{\mathrm{C-O}}_{\mathrm{min}}$, respectively. Fig. S5 shows the relationship between the two quantities for the 199 structures in our data set. In this plot, the systems are colored according to the SAA host, and the points that are part of the identified SG are marked with crosses. For some of the systems, the values of $\Delta d^{\mathrm{C-O}}_{\mathrm{max}}$ and $\Delta d^{\mathrm{C-O}}_{\mathrm{min}}$ are very close. These systems are related to a symmetric chemisorption of the CO_2. This symmetric chemisorption is observed for the adsorption modes η^1 and η^3, as displayed in Fig. S3 (a) and (c).

For the remaining systems, there are no clear correlations between $\Delta d^{\mathrm{C-O}}_{\mathrm{max}}$ and $\Delta d^{\mathrm{C-O}}_{\mathrm{min}}$. These latter systems are related to an asymmetric chemisorption, observed for η^2 mode, as depicted in Fig. S3 (b). The structures associated with the largest $\Delta d^{\mathrm{C-O}}_{\mathrm{max}}$ correspond to an asymmetric η^2 chemisorption mode. Thus, the activation of CO_2 in the considered SAA systems is mainly favored by the asymmetric η^2 chemisorption mode. Indeed, most of the surface sites in the identified SG ($\sigma_{\mathrm{selected}}$) correspond to such asymmetric chemisorption.

S2.3 Analysis of Charge Transfer Between Surface and Chemisorbed CO_2

The charge transfer provided by transition metal surfaces is associated with CO_2 activation.[7] Relying on Mulliken charges computed with the FHI-aims "light" basis set, we explore the relations among the charge in the chemisorbed CO_2 (q^{CO_2}) and the adsorption energy, the OCO angle, and the total C−O bond elongation ($\Delta d^{\mathrm{C-O}}_{\mathrm{Total}} = \Delta d^{\mathrm{C-O}}_{\mathrm{max}} + \Delta d^{\mathrm{C-O}}_{\mathrm{min}}$).[8] These relationships are shown in the plots of Figure S6.

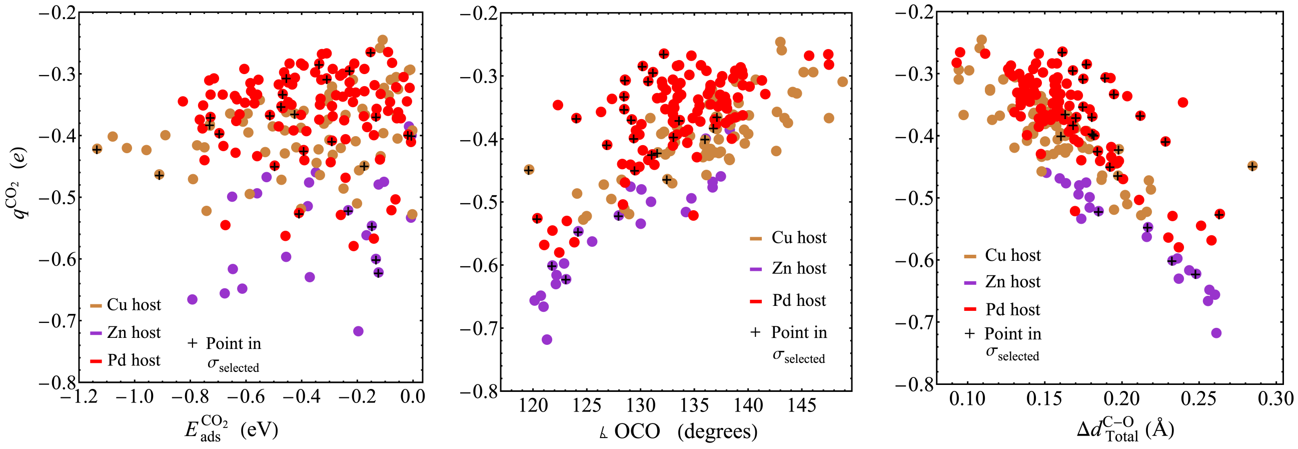

(a) (b) (c)

Fig. S6: Variation of the CO_2 charge (q^{CO_2}) in the chemisorbed structures found on the studied SAAs. Here, we compare q^{CO_2} against three common indicators of activation: (a) $E^{CO_2}_{\mathrm{ads}}$, (b) the OCO angle, and (c) $\Delta d^{\mathrm{C-O}}_{\mathrm{Total}}$.

The host metal providing the larger amounts of charge transfer is Zn. According to Fig. S6 (a), the $E^{CO_2}_{\mathrm{ads}}$ seems to be mostly independent of the charge transferred by the host and no clear trend is noticed. Panel (b) shows a seemingly linear trend between the adsorbate charge and the OCO angle. As expected, the larger the charge transfer the smaller the angle. This is mainly due to the accumulation of charge in the antibonding orbitals ($2\pi_u$) of CO_2.[9] Figure S6 (c) also shows a nearly linear trend between the total bond elongation $\Delta d^{\mathrm{C-O}}_{\mathrm{Total}}$ and the charge. This trend is consistent with the results obtained by Wang et al.[8] In Fig. S5 (b) we analyze the charge transfer by considering the elongations of both C−O bonds separately. Low amounts of charge transfer (high q^{CO_2} values) are associated with shorter elongations. On the other hand, large amounts of charge transfer (low q^{CO_2} values) seem to favor large bond elongations in both symmetric and asymmetric CO_2 chemisorption modes.

S2.4 Adsorption Energies and C−O Bond Elongation: Results for Cu- and Zn-Based SAAs

In the main text, we analyze the role of the surface termination and the SA element in the CO_2 adsorption energy and maximum C−O bond elongation in SAAs based on Pd. Here, we present an analogous analysis for the SAAs based in Cu (Fig. S7) and Zn (Fig. S8) hosts.

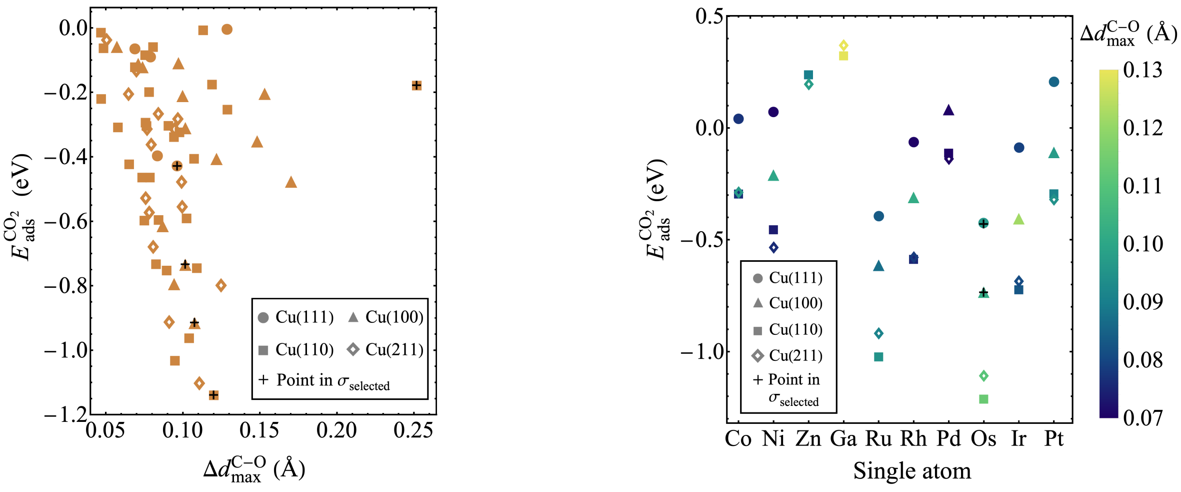

(a) (b)

Fig. S7: (a) The Cu-based SAAs have the structures with the strongest interactions with CO_2. (b) For a given SA, small variations in $\Delta d_{\mathrm{max}}^{\mathrm{C-O}}$ among the different surface terminations are observed. We notice a decrease in the CO_2 interaction with the surface as the periodic table group of the SA increases. This plot only considers bridge sites.

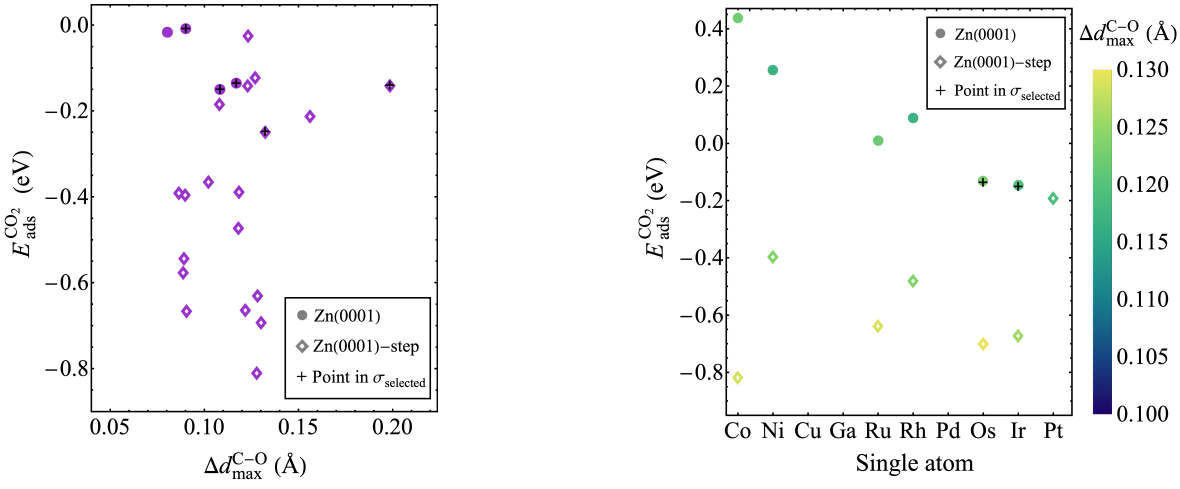

(a) (b)

Fig. S8: (a) From the two studied surfaces in the Zn-based SAAs, the (0001) surfaces only display weak interaction with CO_2. In the case of the stepped surfaces, a SA substitution can provide a fairly wide range of $E_{\mathrm{ads}}^{\mathrm{CO_2}}$. (b) The elongation is not so dependent on the SA as in the Cu and Pd cases, in fact, these values are more stable along all the different chemisorbed structures. Here, the considered sites are ontop (η^3).

In the case of Cu, the range for the $E_{\text{ads}}^{\text{CO}_2}$ is [−1.2, 0 eV] with the (110) and (211) surfaces displaying the stronger binding values. Moreover, the chemisorption of CO_2 in the long bridge of Os@Cu(110) is the structure with the largest elongation ($\Delta d_{\text{max}}^{\text{C-O}} = 0.252$ Å) among all structures in the dataset. Concerning the SA role in the chemisorption (Fig. S7 (b)), the $E_{\text{ads}}^{\text{CO}_2}$ tends to decrease as the group of the atom within a period increases. Although $\Delta d_{\text{max}}^{\text{C-O}}$ does not show a strong trend among the structures, the SAs with the largest elongations are Os and Pt. The limited capability of Ir@Cu SAAs to activate CO_2 might be the reason why this element is not in selected SG (see Table II in the main text). For the SAAs based on Zn, our simulations only found a handful of stable structures for Zn(0001). In fact, Fig. S8 (a) shows how the (0001) is not an appropriate surface to perform the activation of CO_2 as the interaction is extremely weak ($E_{\text{ads}}^{\text{CO}_2} > -0.15$ eV). In the case of the Zn(0001)-stepped surface, the situation is more favorable and the $E_{\text{ads}}^{\text{CO}_2}$ range is [−0.8, 0 eV]. Figure S8 (b) shows how the SA does not seem to have a strong influence on $\Delta d_{\text{max}}^{\text{C-O}}$. However, we note that the number of considered adsorption sites is low relatively low to make general conclusions about SAAs based on this host metal.

S3 Subgroup Discovery (SGD)

S3.1 Brief Description of the Method

In this Section, we provide a concise description of the SGD method.[10,11] Additionally, the NOMAD AI Toolkit[12] contains a detailed tutorial about the use of SGD in the context of catalysis.[13] Let's consider a data set containing a target quantity of interest Y, and many candidate descriptive parameters (or features) φ_i whose values are known for all the samples (data points) of the data set (Fig. S9). The data set is denoted $\widetilde{P}$ to highlight that it is a (typically small) part of the full population P. The values of Y for the full population are unknown. Taking such data set as input, the SGD[10] approach:

a) generates propositions π_i about the φ_i as boolean expressions. For numerical values, these propositions are typically inequalities constraining the values of φ_i to some minimum or maximum values to be determined during the analysis, for instance

$$\pi_1 \equiv \varphi_1 > a \ , \ \pi_2 \equiv \varphi_2 < b \ , \ \dots \ , \ \pi_N \equiv \varphi_N \leq c. \tag{S5}$$

A finite, tractable set of thresholds in these π_i are considered in the SGD analysis and determined through k-means clustering,[11,14] where the number of k is chosen beforehand.

b) defines selectors σ_i as conjunctions of the π_i.

$$\sigma_i = \pi_1 \ \wedge \ \pi_2 \ \wedge \ \dots \tag{S6}$$

These σ_i define SGs within the data set. If a data point follows all the propositions in a selector, the sample belongs to the SG.

c) identifies the selectors corresponding to the most outstanding SGs. These subselections of data maximize a quality function Q

$$Q(SG, \widetilde{P}) = \frac{s(SG)}{s(\widetilde{P})} \cdot u(SG, \widetilde{P}). \tag{S7}$$

Here, the first term is the ratio between the size of the subgroup $s(SG)$ and the size of the data set $s(\widetilde{P})$, also called coverage. This term prevents the selection of too small SGs as they will be statistically irrelevant. The second term is the utility function $u(SG, \widetilde{P})$, which quantifies the usefulness of the SGs. Crucially, $u(SG, \widetilde{P})$ is chosen according to the question to be addressed. For instance, the SGs of interest could be those with either low or high Y values.

The target in our analysis is $\Delta d_{\text{max}}^{\text{C-O}}$ as defined in Eq. 2 in the main text. As we are interested in structures with large $\Delta d_{\text{max}}^{\text{C-O}}$ values, we use the normalized positive mean shift utility function:

$$u(SG, \widetilde{P}) = \frac{\overline{Y}(SG) - \overline{Y}(\widetilde{P})}{Y_{max}(\widetilde{P}) - \overline{Y}(\widetilde{P})}. \tag{S8}$$

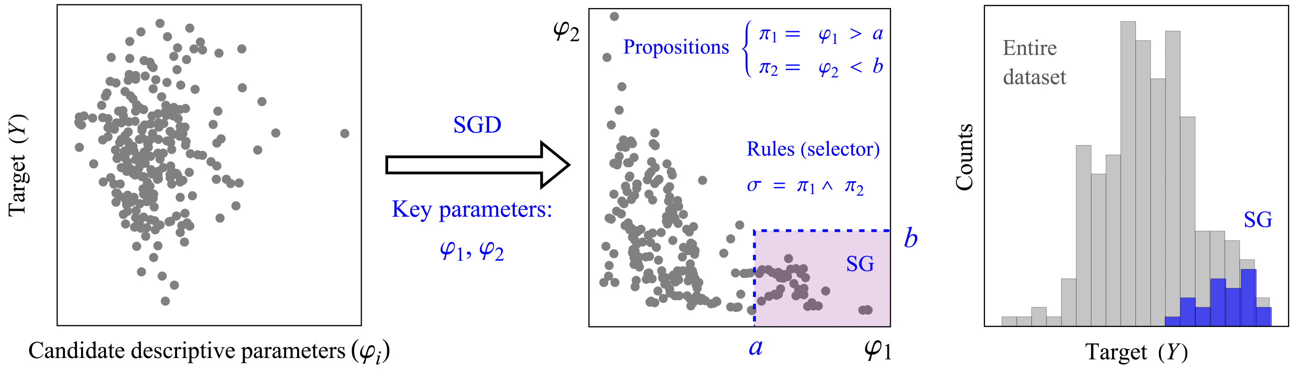

Fig. S9: Starting with a data set containing N physicochemical candidate descriptive parameters (φ_i) and a target quantity of interest Y, *e.g.*, a materials property, SGD searches along the φ_i-space for regions where the subgroups (SGs) with exceptional distributions of Y are located. Then, SGD provides selectors σ_i as conjunctions of propositions about key φ_i, out of all the offered ones, characterizing the outstanding SGs. We identify these selectors as "rules" that can be used to obtain physical insights and to screen for new materials outside the training data set. In this example, the uncovered rules only depend on parameters φ_1 and φ_2, but in practice, the selectors may involve many features.

In Eq. S8, $\overline{Y}(\widetilde{P})$ and $Y_{max}(\widetilde{P})$ represent the mean and maximum values of $\Delta d_{\text{max}}^{\text{C-O}}$ distribution over the data set. $\overline{Y}(SG)$ refers to the mean value of Y in the SG. By using this utility function, SGs with high mean values of $\Delta d_{\text{max}}^{\text{C-O}}$ are favored.

S3.2 Additional Details on the Candidate Descriptive Parameters

To derive the physicochemical parameters used as candidate descriptive parameters in our SGD analysis (Table I in the main text), we collected parameters reflecting free-atom properties of the elements in the alloy. The electron affinity and ionization potential are taken from experimental measurements for the free atoms.[15] The *s*-, *p*-, *d*-, and valence-orbital radii were taken from the atomic-features-package.[16] These free-atom radii were calculated with FHI-aims, DFT-PBEsol[17] and the "very tight" basis set. They correspond to the radii at which the electron density has the maximum value in an isolated atom of a given element. The following geometrical parameters characterizing the surface sites are the usual coordination number (CN) and the generalized-CN (gen-CN).[18] Instead of counting the atoms in the first coordination sphere as 1, the generalized-CN is a metric including a weighted CN_i for each neighbor i. We define these neighbors as the atoms that are within a certain cutoff distance from the atoms in the adsorption site. This cutoff distance is taken as the closest interatomic distance of atoms in the bulk of the host (Cu: 2.53 Å, Zn: 2.61 Å, Pd: 2.75 Å). The gen-CN is defined as

$$\text{gen-CN (atom/site)} = \frac{1}{\text{CN}_{\text{max}}} \sum_i \text{CN}_i, \quad \text{(S9)}$$

where CN_{max} is the maximum coordination of an equivalent atom/site ensemble in bulk. Thus, gen-CN provides a more general description of the geometrical environment of a given surface site.

S3.3 Determination of Parameters for SG Search

The SGD studies were performed with realKD[11,19] and using a Monte-Carlo-based search algorithm.[20] Two parameters need to be chosen to perform the SGD search with this algorithm: a) the number of clusters (k) utilized to create the propositions (see Subsection 3.1), and b) the number of seeds (N_{seeds}) used to initialize the stochastic sampling of σ. The stochastic search algorithm does not guarantee finding the optimal SGD solution. Thus, we need to understand which combination of k and N_{seeds} values provide stable (converged) results. We tested the influence of k on the SGD studies, by running the algorithm with k = 6, 8, 10, 12, 14, 16, and 20. In

principle, setting a N_{seeds} value as large as possible, *e.g.* several million, should provide a converged result for a given k value. Nonetheless, using such a large value for N_{seeds} is computationally unfeasible. Therefore, we analyzed the influence of N_{seeds} on the SGD results by using 100-, 200-, 300-, 500-thousand, and 1 million seeds. We perform a small statistical analysis by running SGD three times with each of the possible k, N_{seeds} settings. Finally, we compared the results change for the particular k, N_{seeds} pairs.

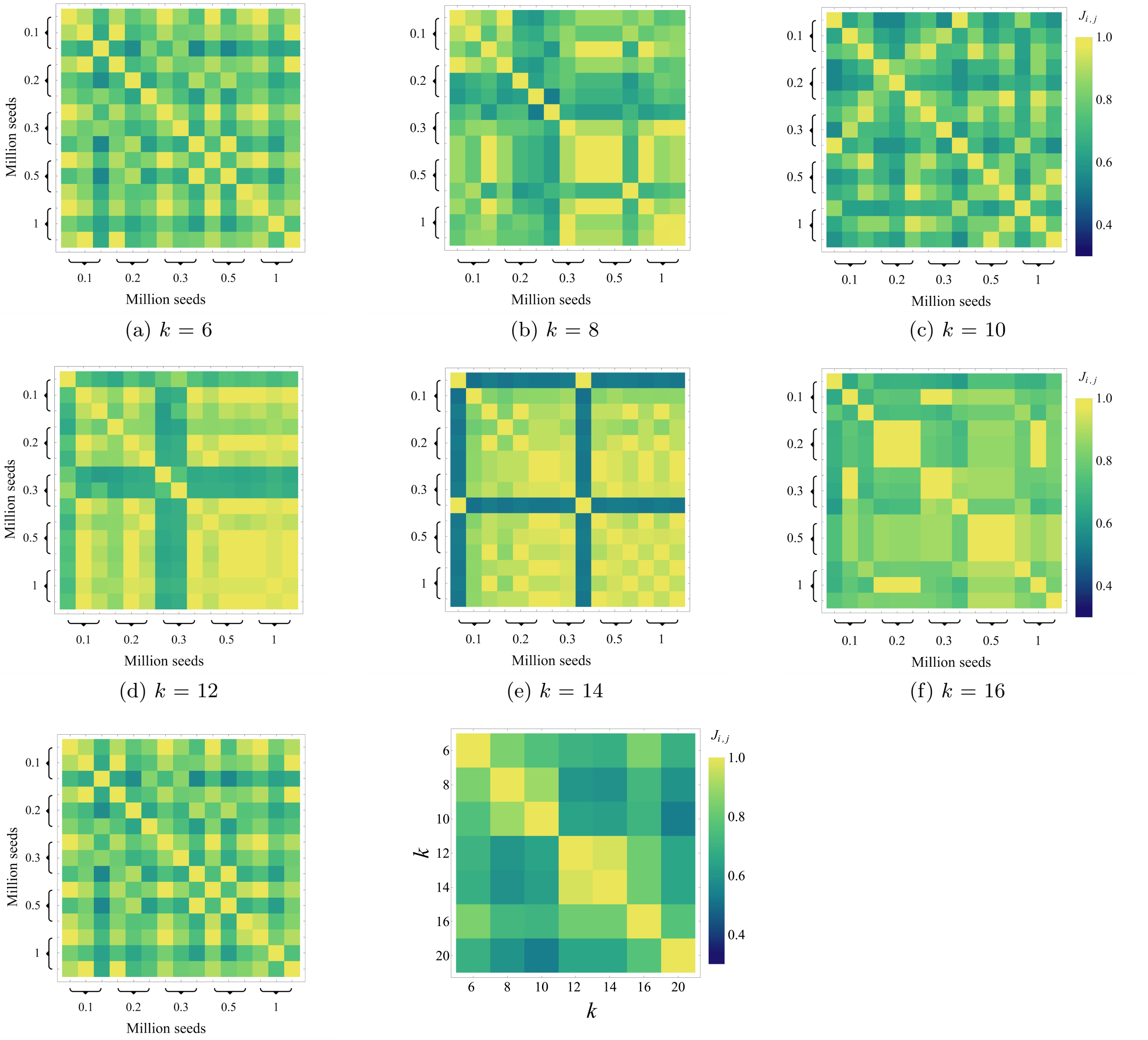

(a) $k = 6$ (b) $k = 8$ (c) $k = 10$

(d) $k = 12$ (e) $k = 14$ (f) $k = 16$

(g) $k = 20$ (h) k comparisson

Fig. S10: (a-g) Matrix representations of the Jaccard similarity index (J) for different numbers of k-means and random seeds used during the Monte Carlo generation of selectors (σ_i). Three runs per N_{seeds} were performed, and the evaluation of J was based on the SGs maximizing $Q(SG, \widetilde{P})$. The high similarity region when k=12 and $N_{\text{seeds}} \geq$ 500-thousand led us to choose these settings to search outstanding SAAs for the CO_2 activation. (h) J values among the tested k values. The comparison is made for a $\sigma_{max(Q)}$ obtained with 500-thousand N_{seeds}.

Our comparison between two SGD evaluations is based on the data points associated with the SG maximizing

the quality function, $Q(SG, \widetilde{P})$, termed $\sigma_{max(Q)}$. We used the Jaccard index (J) as a metric of the similarity between two SGs i and j:[21]

$$J(SG_i, SG_j) = \frac{\mid SG_i \cap SG_j \mid}{\mid SG_i \cup SG_j \mid}. \tag{S10}$$

J is the ratio between the number of data points in the intersection and the union of the SGs i and j. Hence, if the SGs have the same data points, the index value is equal to 1, and when the compared SGs have a $J = 0$, these do not share any data points. The matrices in Fig. S10 show the value of $J(SG_i, SG_j)$ (as color code) for the SGs $\sigma_{max(Q)}$ obtained using different k-means and N_{seeds}. In each matrix, the x and y axes show the value of the used seeds in millions. As three runs per each N_{seeds} were performed, the brackets in the axes point to these three independent runs.

The fact that these J values are not always equal to 1.0 reflects the variability of the solutions provided by the stochastic search algorithm. The J index calculated between SGs obtained using the same number of k and N_{seeds} can be within the range [0.48, 1.0], with small fluctuations for the minimal value of the index (being 0.48 the lowest among all the considered k values). For k =6 (Fig. S10(a)), J oscillates along all the considered N_{seeds}. The latter implies that, even if we use 1 million seeds, there is no evidence of reaching a converged result. This situation is similar for k =8, 10, 16, and 20 (Fig. S10(b,c,f,g)), where the J values denote the lack of convergence in the SGD solutions. However, the matrices for k = 12, 14, show an almost converged region for $N_{\text{seeds}} > 500{,}000$. For these combination settings, J is in the range [0.9, 1.0]. Therefore, the SGs $\sigma_{max(Q)}$ obtained through different runs are almost identical. Figure S10(h) compares $\sigma_{max(Q)}$ for SGs obtained by 500-thousand seeds and along the considered k values. A fairly strong fluctuation in J is noticeable among most of the k's. Nevertheless, the results for k =12, 14 find basically the same SG $\sigma_{max(Q)}$ ($J > 0.97$.) Based on these analyses, we conclude that the SGs obtained with k=12 and $N_{\text{seeds}} \geq$ 500-thousand are converged. Therefore, we used these settings in our SGD studies.

S3.4 Rules Associated with the SG with Maximum Quality-Function Value

In our Pareto front analysis (see Results Section in the main text), we found a set of optimal solutions associated with different coverage-utility tradeoffs of the quality function ($Q(SG, \widetilde{P})$). The main goal of our study was to find SGs of SAAs surface sites with an exceptional capability to activate CO_2. For this reason, in the main text, we focus the discussion on the SG with the highest identified utility function (σ_{selected}). Here, we provide further details on the identified SG that presents the maximum quality-function value ($\sigma_{\max(Q)}$), *i.e.*, the SG that would be obtained with the standard SGD approach (without a Pareto-front analysis). The rules characterizing this SG are the following:

$$\sigma_{\max(Q)} \;:\; \text{EA}_{\text{SA}} \leq 1.565\,\text{eV} \;\wedge\; \text{PE}_{\text{SA}} \geq 1.73\,\text{eV} \;\wedge\; \text{IP}_{\text{site}} \geq 8.068\,\text{eV} \;\wedge\; \text{CN} \leq 6.5 \tag{S11}$$

The electron affinity (EA_{SA}) and Pauling electronegativity (PE_{SA}) of the SA, and the ionization potential (IP_{site}) and coordination number (CN) of the surface site as the key parameters describing SG. If we compare these rules with the ones for σ_{selected}, it is possible to notice certain similarities. For instance, the electron affinity and Pauling electronegativity appear in both sets of rules, but in one case these terms refer to the SA and, in the second one, to the surface site. The geometric environment is also relevant as σ_{selected} provides a threshold for the gen-CN, while $\sigma_{\max(Q)}$ considers the usual CN. In fact, from the 26 data points in σ_{selected}, 25 of them are contained in $\sigma_{\max(Q)}$. The surface sites in $\sigma_{\max(Q)}$ are listed in Table S10. According to the CN $\leq$ 6.5 threshold, these surface sites do not contain one-atom sites. A total of 7 SAs can be seen in this SG: Co, Ni, Cu, Ru, Rh, Os, and Ir. In the case of σ_{selected}, we only find Os and Ir. It could be possible to use these rules to screen new SAAs. Nonetheless, we should expect less effective CO_2 activation for the predicted materials.

Table S10: In the main text, we focus on the SG maximizing the utility function (σ_{selected}), *i.e.*, the most exceptional subset in the Pareto front of SGD solutions. Here, we show the surface sites and SAs belonging to the SG maximizing the quality function ($\sigma_{\max(Q)}$). The larger number of members in $\sigma_{\max(Q)}$ shows how this SG provides not only a more general solution but also a less useful one when searching for outstanding materials for CO_2 activation.

Host	Surface	Sites	SAs
Cu	111	Bridge, hcp	Os, Ir
	100	Bridge, hollow	
	110	Short bridge, long bridge, 4-fold hollow	
	211	Bridge2-s	
Zn	0001	Ontop, bridge	Ru, Os, Ir
	0001-step	Top-s, Bridge1-s, Bridge2-s, Bridge3-s, fcc-s	Co, Ni, Ru, Rh, Os, Ir
Pd	111	Bridge	Co, Os, Ir
	100	Ontop, bridge	
	110	Short bridge, long bridge, 4-fold hollow, ontop	Co, Ni, Cu, Ru, Rh, Os, Ir
	211	Top-s, bridge1-s, Bridge2-s, bridge3-s, fcc-s,	Cu, Os, Ir

S4 Stability of the SAAs

S4.1 Formation Energy Analysis

As an initial proxy for their synthesizability and stability, we evaluate the formation energy of the SAAs (E_f^{SAA})[22] considered in this work as:

$$E_f^{\text{SAA}} = (E_{\text{slab}}^{\text{SAA}} + E_{\text{bulk}}^{\text{host}}) - (E_{\text{slab}}^{\text{host}} + E_{\text{bulk}}^{\text{SA}}). \quad \text{(S12)}$$

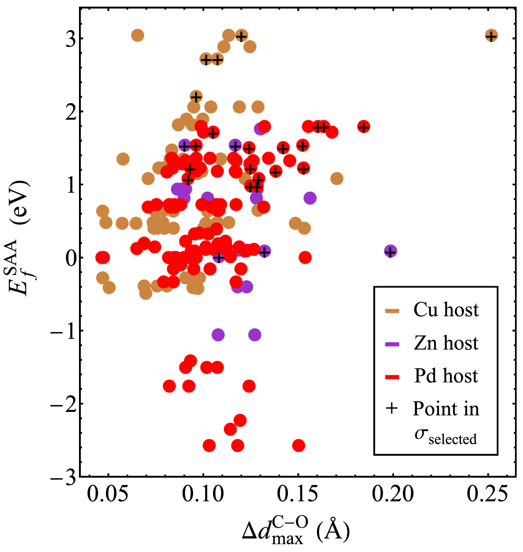

Fig. S11: Formation energy values according to the C−O elongation in the CO_2 activation data set. The structures in σ_{selected} belong to high E_f^{SAA} surfaces.

Table S11: Zn-based SAAs formation energies. FHI-aims "light" basis set.

	$E_f^{\text{Zn-SAA}}$ (eV)	
SA	(0001)	(0001)-step
Co	0.813	0.812
Ni	0.129	0.097
Cu	0.119	0.082
Ga	0.063	−0.117
Ru	0.689	0.941
Rh	−0.485	−0.395
Pd	−0.932	−0.845
Ag	−0.004	−0.068
Os	1.536	1.760
Ir	0.014	0.085
Pt	−1.123	−1.061
Au	−0.677	−0.644

In eq. S12, $E_{\mathrm{slab}}^{\mathrm{SAA}}$ and $E_{\mathrm{slab}}^{\mathrm{host}}$ are the energies of the SAA and host model slabs, respectively. $E_{\mathrm{bulk}}^{\mathrm{host}}$ and $E_{\mathrm{bulk}}^{\mathrm{SA}}$ are the energies per atom in the bulk for the host and SA. Due to the impossibility of modeling isolated surface atoms, Eq. S12 provides a way to compare an energy difference between an equivalent number of atoms, *i.e.*, the same number of host atoms and the one SA. Thus, a negative E_f^{SAA} value implies that the substitution of one atom in the host surface by the SA generates a more stable system than the pristine host surface.

We note that the evaluation of $E_{\mathrm{bulk}}^{\mathrm{host}}$ and $E_{\mathrm{bulk}}^{\mathrm{SA}}$ requires the optimization of the hosts and SAs lattices. As FHI-aims could not perform spin-polarized stress computations for meta-GGA functionals, optimizing the Co and Ni lattice with mBEEF was not possible. Thus, to keep consistency during the evaluation of E_f^{SAA} in the SAAs containing Co and Ni, we used spin-polarized DFT-PBEsol calculations. Tables S11 and S12 provide the computed E_f^{SAA} for all the Cu-, Zn-, and Pd- SAAs. From these data, we notice that 46% of the considered SAAs have a favorable formation energy. Nonetheless, some of the stable systems contain Ag and Au, alloys where CO_2 was not activated.

Table S12: Cu-, Pd-based SAAs formation energies. FHI-aims "light" basis set.

	$E_f^{\text{Cu-SAA}}$ (eV)					$E_f^{\text{Pd-SAA}}$ (eV)			
SA	(111)	(100)	(110)	(211)	SA	(111)	(100)	(110)	(211)
Co	0.992	1.099	1.228	1.152	Co	0.320	0.395	0.114	0.317
Ni	0.257	0.403	0.477	0.397	Ni	0.322	0.330	0.094	0 .227
Zn	−0.577	−0.538	−0.606	−0.550	Cu	−0.157	−0.100	−0.337	−0.157
Ga	−0.994	−0.844	−0.880	−0.891	Zn	−1.550	−1.414	−1.764	−1.508
Ru	1.467	1.814	2.061	1.892	Ga	−2.348	−2.226	−2.578	−2.349
Rh	0.205	0.472	0.636	0.466	Ru	1.174	1.278	1.324	1.359
Pd	−0.487	−0.442	−0.384	−0.491	Rh	0.649	0.723	0.690	0.729
Ag	−0.003	−0.147	−0.199	−0.200	Ag	−0.329	−0.376	−0.585	−0.418
Os	2.209	2.720	3.040	2.890	Os	1.502	1.540	1.712	1.798
Ir	0.643	1.080	1.350	1.177	Ir	0.980	1.077	1.224	1.182
Pt	−0.681	−0.421	−0.276	−0.414	Pt	0.010	0.120	0.193	0.150
Au	−0.624	−0.588	−0.579	−0.636	Au	−0.528	−0.501	−0.544	−0.491

Figure S11 shows the formation energies (y-axis) of the SAAs with respect to the maximum elongation of the C−O bond (x-axis) in the chemisorbed CO_2 structures within our data set. The crosses in the Figure point to the data points belonging to $\sigma_{\mathrm{selected}}$ (Ir and Os SAA surface sites) and are located in the high E_f^{SAA} region. Among the points in $\sigma_{\mathrm{selected}}$, the surface sites on the Ir@Zn(0001) and Ir@Zn(0001)-step provide a good reactivity-stability balance with a near zero E_f^{SAA} and $\Delta d_{\mathrm{max}}^{\mathrm{C-O}}$ in the range [0.11,0.13 Å]. Moreover, although not part of $\sigma_{\mathrm{selected}}$, some SAAs based in Cu and Pd hosts, also provide promising reactivity-stability tradeoffs. For instance, Rh@Cu(100) or Pt@Pd(111). We stress that under operation conditions (high temperature and pressure), the surface of the alloys can suffer reconstruction. Hence, this analysis of the SAAs formation energy provides only an initial step to understanding the stability of these materials.

S4.2 Segregation Energies in the SAAs

Metal segregation in alloy materials is a well-known phenomenon.[23] In the case of SAAs, segregation could play a major role in their stability, and therefore, their virtual application as catalysts.[24] To provide a second criterion aiming to model the stability of the SAAs considered within our work, we compute the SA's segregation energy (ΔE_{seg}) to the host bulk in 84 surfaces.

$$\Delta E_{\mathrm{seg}} = E_{\mathrm{sublayer}} - E_{\mathrm{SAA}}, \tag{S13}$$

in the previous equation, E_{sublayer} corresponds to the energy of a slab where a given SA has substituted an atom in the third layer. The second term E_{SAA} is the energy of a slab where a SA substituted an atom of the top layer. If E_{sublayer} displays a value higher than zero, the SA is stable at the surface. When the E_{sublayer} is lower than zero, the segregation of the SA to subsurface layers is thermodynamically preferred. We plot the values of ΔE_{seg} for the considered surfaces in Fig. S12.

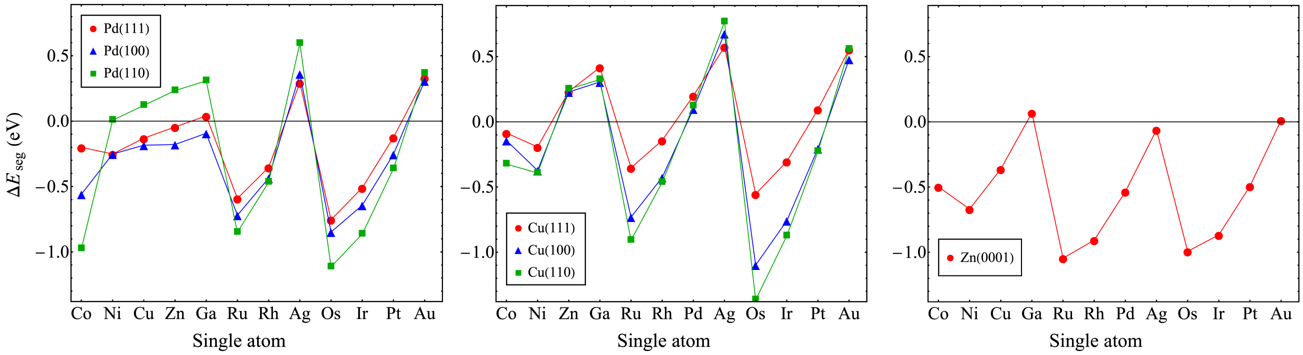

(a) (b) (c)

Fig. S12: Segregation energy (ΔE_{seg}) values for 84 out of the 120 SAA surfaces considered in this study. A positive ΔE_{seg} is associated with a SA stable at the surface layer. (a) SAAs based on Pd. (b) SAAs based on Cu. (c) SAAs based on Zn.

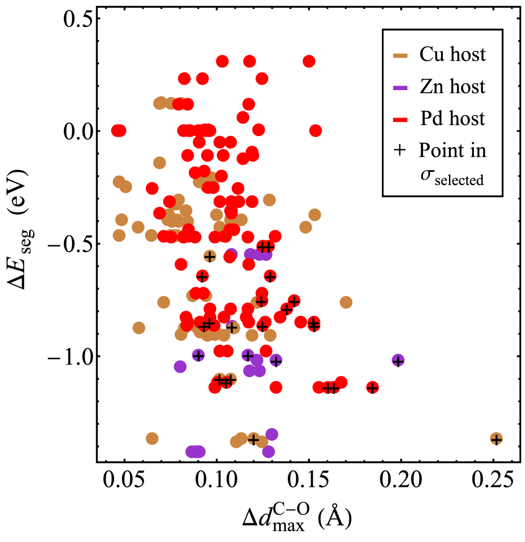

Fig. S13: Segregation energy variation with respect to the C−O elongation. The points in $\sigma_{\mathrm{selected}}$ are associated with SAAs where the SA segregation to subsurface layers is favored.

As in the analysis of E_f^{SAA}, the SAAs showing the highest values of $\Delta d_{\mathrm{max}}^{\mathrm{C-O}}$ are the most unstable, *i.e.*, the segregation of the SA to the bulk is energetically preferred ($\Delta E_{\mathrm{seg}} < 0$). Nonetheless, the SA is stable at the surface in 30% of the SAAs based in Pd. The percentage of SAs stable at the surface is ever higher in Cu as host with 40%. Finally, Zn has the lowest amount of SAs stable at the surface, with only 17% of the tested systems. We also note a seemingly periodic pattern in Fig. S12. If we center our attention on a given period, ΔE_{seg} tends to increase with the atomic number of the SA. Therefore, stable SAAs based on Cu, Zn, and Pd might be likely with SAs located on the right side of the periodic table.

The variation of ΔE_{seg} against the C−O bond elongation is plotted in Fig.S13. We notice how the surface sites selected by the SG rules belong to SAAs where the SA segregation to subsurface layers is an energetically favored process ($\Delta E_{\mathrm{seg}} < 0$). Nonetheless, some surface sites display a seemingly good compromise between the SAA stability and large $\Delta d_{\mathrm{max}}^{\mathrm{C-O}}$.

References

[1] Blum, V.; Gehrke, R.; Hanke, F. et al. *Computer Physics Communications* **2009**, *180*, 2175–2196.

[2] Kabalan, L.; Kowalec, I.; Catlow, C. R. A. et al. *Physical Chemistry Chemical Physics* **2021**, *23*, 14649–14661.

[3] Wellendorff, J.; Lundgaard, K. T.; Jacobsen, K. W. et al. *The Journal of Chemical Physics* **2014**, *140*, 144107.

[4] Schmidt, P. S.; Thygesen, K. S. *The Journal of Physical Chemistry C* **2018**, *122*, 4381–4390.

[5] Miyazaki, R.; Faraji, S.; Levchenko, S. V. et al. *Catal. Sci. Technol.* **2024**, *14*, 6924–6933.

[6] Chan, C. T.; Bohnen, K. P.; Ho, K. *Physical Review B* **1993**, *47*, 4771.

[7] Freund, H.-J.; Roberts, M. W. *Surface Science Reports* **1996**, *25*, 225–273.

[8] Wang, S.-G.; Liao, X.-Y.; Cao, D.-B. et al. *The Journal of Physical Chemistry C* **2007**, *111*, 16934–16940.

[9] Álvarez, A.; Borges, M.; Corral-Pérez, J. J. et al. *ChemPhysChem* **2017**, *18*, 3135–3141.

[10] Wrobel, S. An Algorithm for Multi-relational Discovery of Subgroups. European symposium on principles of data mining and knowledge discovery. 1997; pp 78–87.

[11] Goldsmith, B. R.; Boley, M.; Vreeken, J. et al. *New Journal of Physics* **2017**, *19*, 013031.

[12] Sbailò, L.; Fekete, Á.; Ghiringhelli, L. M. et al. *npj Computational Materials* **2022**, *8*, 250.

[13] Foppa, L.; Sutton, C.; Ghiringhelli, L. M. et al. *ACS Catalysis* **2022**, *12*, 2223–2232.

[14] MacQueen, J., et al. Some methods for classification and analysis of multivariate observations. Proceedings of the fifth Berkeley symposium on mathematical statistics and probability. 1967; pp 281–297.

[15] National Center for Biotechnology Information. Periodic Table of Elements. `https://pubchem.ncbi.nlm.nih.gov/periodic-table`.

[16] Atomic-features-package. `https://gitlab.mpcdf.mpg.de/nomad-lab/ai-toolkit/packages/atomic-features-package`.

[17] Perdew, J. P.; Ruzsinszky, A.; Csonka, G. I. et al. *Physical review letters* **2008**, *100*, 136406.

[18] Calle-Vallejo, F.; Martínez, J. I.; García-Lastra, J. M. et al. *Angewandte Chemie International Edition* **2014**, *53*, 8316–8319.

[19] realKD - open-source Java library. `https://bitbucket.org/realKD/realkd/wiki/Home`.

[20] Boley, M.; Lucchese, C.; Paurat, D. et al. Direct local pattern sampling by efficient two-step random procedures. 17th ACM SIGKDD International conference on knowledge discovery and data mining. 2011; pp 582–590.

[21] Foppa, L.; Scheffler, M. *arXiv preprint arXiv:2403.18437* **2024**,

[22] Behrendt, D.; Banerjee, S.; Clark, C. et al. *Journal of the American Chemical Society* **2023**, *145*, 4730–4735.

[23] Ruban, A.; Skriver, H. L.; Nørskov, J. K. *Physical Review B* **1999**, *59*, 15990.

[24] Papanikolaou, K. G.; Darby, M. T.; Stamatakis, M. *The Journal of Physical Chemistry C* **2019**, *123*, 9128–9138.
